# Cooperativity and Rapid Evolution of Cobound Transcription Factors in Closely Related Mammals

**DOI:** 10.1016/j.cell.2013.07.007

**Published:** 2013-08-01

**Authors:** Klara Stefflova, David Thybert, Michael D. Wilson, Ian Streeter, Jelena Aleksic, Panagiota Karagianni, Alvis Brazma, David J. Adams, Iannis Talianidis, John C. Marioni, Paul Flicek, Duncan T. Odom

**Affiliations:** 1 Cancer Research UK Cambridge Institute, Li Ka Shing Centre, University of Cambridge, Cambridge CB2 0RE, UK; 2 European Molecular Biology Laboratory, European Bioinformatics Institute, Wellcome Trust Genome Campus, Hinxton, Cambridge CB10 1SD, UK; 3 Genetics & Genome Biology Program, Hospital for Sick Children (SickKids) and Department of Molecular Genetics, University of Toronto, 101 College Street, East Tower, Toronto, ON M5G 1L7, Canada; 4 Department of Genetics, University of Cambridge, Cambridge CB1 3QA, UK; 5 Cambridge Systems Biology Centre, University of Cambridge, Cambridge CB2 1QR, UK; 6 Biomedical Sciences Research Center Alexander Fleming, 16672 Vari, Greece; 7 Wellcome Trust Sanger Institute, Wellcome Trust Genome Campus, Hinxton, Cambridge CB10 1SA, UK

## Abstract

To mechanistically characterize the microevolutionary processes active in altering transcription factor (TF) binding among closely related mammals, we compared the genome-wide binding of three tissue-specific TFs that control liver gene expression in six rodents. Despite an overall fast turnover of TF binding locations between species, we identified thousands of TF regions of highly constrained TF binding intensity. Although individual mutations in bound sequence motifs can influence TF binding, most binding differences occur in the absence of nearby sequence variations. Instead, combinatorial binding was found to be significant for genetic and evolutionary stability; cobound TFs tend to disappear in concert and were sensitive to genetic knockout of partner TFs. The large, qualitative differences in genomic regions bound between closely related mammals, when contrasted with the smaller, quantitative TF binding differences among *Drosophila* species, illustrate how genome structure and population genetics together shape regulatory evolution.

## Highlights

• Earliest steps of regulatory evolution in mammals captured using five mouse species
• Interspecies differences in TF binding are rarely caused by DNA variation in motifs
• Cobound TFs change their genomic binding cooperatively in closely related mammals
• Genetic knockouts revealed the extent of cooperative stabilization in TF binding clusters

## Graphical Abstract

**Figure:**
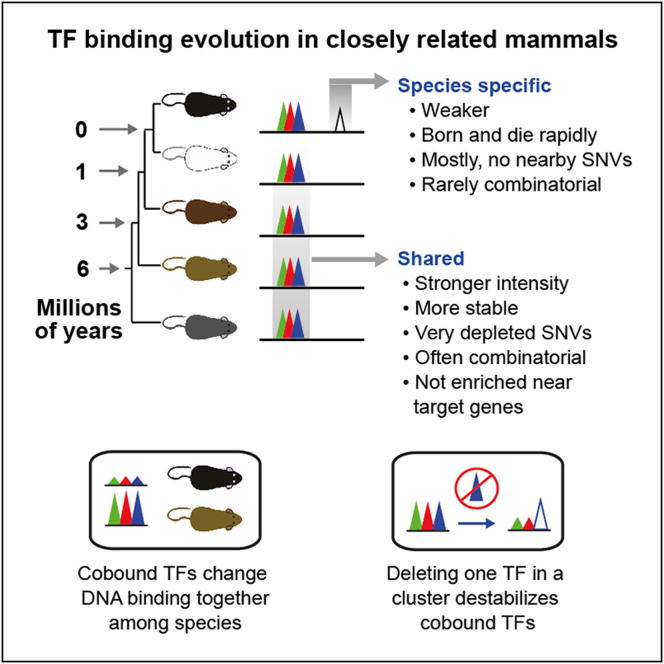

## Teaser

Microevolutionary mechanisms create different transcription factor binding patterns between mammals, shedding light on the regulatory mechanisms partially underlying speciation.

## Introduction

The phenotypic differences observed both among different individuals within one species and between closely related species are often the result of genetic differences within regulatory regions (Stone and Wray, 2001; Wray, 2007). These regulatory regions are bound by tissue-specific transcription factors (TFs) to control complex gene expression phenotypes (Bradley et al., 2010; ENCODE, 2012; Zinzen et al., 2009).

A typical higher eukaryotic TF binds tens to hundreds of thousands of DNA sites and yet may directly control only a few hundred genes (Biggin, 2011). Studies in *Drosophila* suggest that much of this widespread TF binding represents low occupancy, functionally neutral interactions (Bradley et al., 2010; Fisher et al., 2012; MacArthur et al., 2009) that are driven thermodynamically by the relatively high concentrations of TF proteins in nuclei (Lin and Riggs, 1975). Indeed, most tissue-specific TFs bind short, somewhat degenerate DNA sequences that facilitate widespread genomic binding (Jolma et al., 2013), often in clusters that contain multiple different TFs (e.g., combinatorially) (Bradley et al., 2010; ENCODE, 2012; Kvon et al., 2012; MacArthur et al., 2009; Biggin, 2011). Clustered TF binding appears to result in large part from indirect cooperativity to open chromatin regions, as opposed to direct TF-TF protein interactions (Kaplan et al., 2011; Miller and Widom, 2003; Mirny, 2010). For binding sites within a nucleosome-length distance, each TF contributes partially to a competitive displacement of specific nucleosomes by indirect collaboration with other TFs, mutually aiding each others’ binding to DNA. TFs within a cluster can have different regulatory roles depending on their motif strength and ability to compete with nucleosomes (Zinzen et al., 2009). In such a scenario, TF binding would be determined not only by the presence and strength of DNA motifs but also by the cobinding of other TFs to open a DNA-binding region.

Although some studies have shown that TF binding can persist in the absence of sequence constraint (Piano et al., 1999; Ludwig et al., 2000), strong, combinatorial TF binding is thought to most often occur preferentially near target genes at genetic sequences that show evidence of high sequence constraint (He et al., 2011b). In contrast, poorly bound sequences are less constrained and do not drive reporter gene expression (Fisher et al., 2012). This model for transcriptional regulation predicts that strong and functional TF binding will be under greater selective pressure, and thus the protein-DNA contact itself should be preferentially maintained during evolution—particularly in closely related species and possibly by positive selection (He et al., 2011a). Comparison of one developmental TF (*Twist*) in fruit fly embryos from multiple species in a single genus indicated high conservation of TF binding, which was found to be greatest near direct target genes (He et al., 2011b). A similar analysis of the binding of six TFs in embryos from two closely related *Drosophila* species found that most TF binding differences are quantitative (e.g., subtle alterations in TF binding strength) and are rarely complete gains or losses (Bradley et al., 2010). Furthermore, in flies, TF binding differences between species are highly correlated when they occur in combinatorial clusters, which are preferentially maintained between species and may be linked to chromatin accessibility via binding of the TF *vfl* (also known as *Zelda*) (Bradley et al., 2010; Harrison et al., 2011; Nien et al., 2011).

In contrast, the microevolutionary mechanisms that result in differences in TF binding among closely related mammals have not been studied in detail. Mammalian similarities with other animal lineages include the fact that TFs bind predominantly in a combinatorial manner in genetically heterogeneous human cell lines (Reddy et al., 2012; see also Odom et al., 2006). Some TF binding differences between alleles were associated with single nucleotide variations (SNVs) at bound regions, but most allelic differences were not associated with underlying sequence differences (Reddy et al., 2012; see also Kasowski et al., 2010; McDaniell et al., 2010; Spielman et al., 2007; Spivakov et al., 2012). Also similar to the case in flies (Li et al., 2011), open chromatin and TF cobinding can help direct de novo binding of the induced glucocorticoid receptor (Biddie et al., 2011; John et al., 2011).

Despite the many similarities between vertebrate and insect gene regulation, important differences in TF binding evolution have been observed. First, a small proportion of human TF binding events were found to be shared between human and chicken (<2% for CEBPA), whereas apparently more distant *Drosophila* species show almost no changes in TF binding (He et al., 2011b). Second, TF binding events occurring near direct target genes are only modestly more likely to be shared between mouse and human when compared with random TF binding, most of which is likely functionally neutral (Kunarso et al., 2010; Schmidt et al., 2010). Third, human regions strongly bound by TFs do not appear to be preferentially conserved in mice (Schmidt et al., 2010). It is not yet known how these observations relate to shorter evolutionary timescales (e.g., within a given order), but a quantitative understanding of the first steps in TF binding evolution in closely related mammals would help to answer important questions, including the following: are there particular types of binding sites more robust to evolutionary changes? Do they have identifiable molecular characteristics? Is there a direct (or perhaps causal) relationship between genetic divergence and TF binding divergence? How are the sequence variations near binding sites translated into differences in TF occupancy?

To address these questions, we have generated quantitative, in vivo TF occupancy data for three tissue-specific TFs (HNF4A, CEBPA, and FOXA1) in livers from five closely related mice, four of whose genomes have been recently reported (Keane et al., 2011), and rat. Our experiments revealed the rate at which differences in TF binding accumulate in mammals with high accuracy, established the relative contribution of sequence variations toward TF binding occupancy differences, and revealed coordinated changes in TF binding intensities that occur within cobound TF clusters. Finally, by performing additional TF binding experiments in genetically engineered mice lacking either HNF4A or CEBPA, we were able to compare the genetic robustness and cooperativity of clusters of TF binding sites with their evolutionary stability.

## Results

All data have been deposited in ArrayExpress with accession numbers E-MTAB-1414 for mouse and E-MTAB-1415 for rat. The methods are described in the Extended Experimental Procedures, organized by their appearance in the Results.

### Determination of TF-Bound Regions in Five Closely Related Mammals

**Figure 1:**
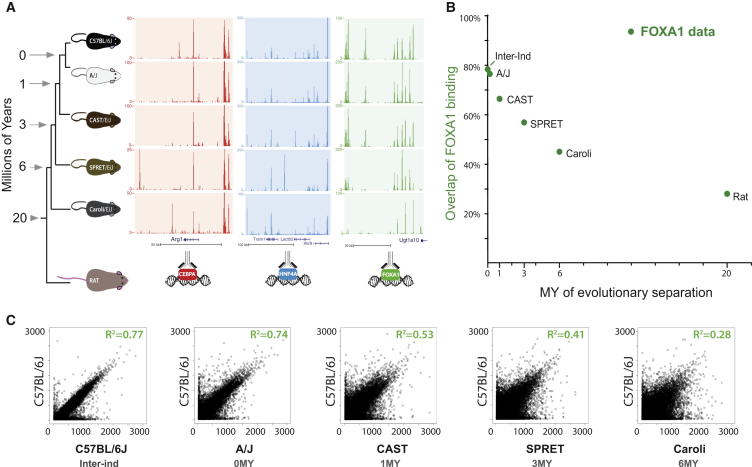
Rate of Accumulation of TF Binding Occupancy Differences between Closely Related Mammals within One Order (A) To assess the rate at which TF binding differences accumulate, we identified and compared the global in vivo binding of FOXA1, CEBPA, and HNF4A in livers of six closely related rodents ranging in evolutionary separation from 1 to 20 MY. Examples of both shared and species-specific TF binding locations are indicated at representative loci. (B) The fraction of FOXA1 binding found at homologous locations when comparing C57BL/6J and other rodent species (*y* axis) is plotted against the evolutionary distance between species in millions of years (*x* axis). (C) FOXA1 binding intensities were compared across the entire genome within and between mouse species. TF binding profiles between individuals within the same species (C57BL/6J) showed a high correlation (green inset, R^2^ = 0.77), which decreased with increasing evolutionary distance. See also Figures S1 and S2.

**Figure S1:**
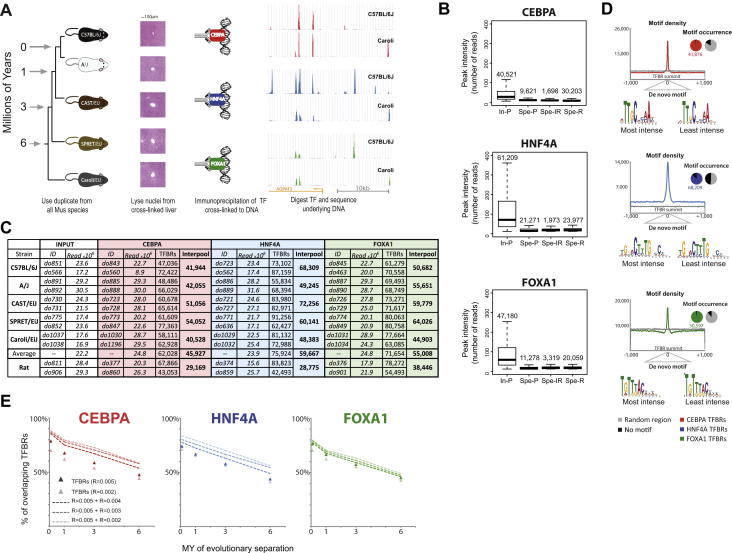
Quality Evaluation of the In Vivo TF Binding Data Assayed in Five Mouse Species, Related to Figure 1 (A) ChIP followed by high-throughput sequencing (ChIP-seq) of CEBPA, HNF4A and FOXA1 in C57BL/6J, A/J, CAST/EiJ, SPRET/EiJ, and Caroli/EiJ mice showing their phylogenetic relationship and H&E stained livers for each species. (B) Intensity distribution of peaks called for final TFBR sets in C57BL/6J: the Inter-pool (In-P)) compared to peaks present in the replicates but excluded from the final set by (i) pooling samples (Spe-P), (ii) overlapping inter-replicate with pool (Spe-IR) or (iii) combining replicates (Spe-R). (C) Summary table of genomic background (Input) and ChIP-seq data sets for CEBPA, HNF4A and FOXA1 duplicate in C57BL/6J, A/J, CAST/EiJ, SPRET/EiJ, and Caroli/EiJ, and rat included in the subsequent analysis. Listed are sequencing run identifiers, the number of reads sequenced, the number of peaks called by SWEMBL in both replicates, and the final number of TFBRs in the Inter-pool used for all downstream analyses. The average TFBR numbers for the five mouse species are also shown in a separate row. (D) Motif analysis of TFBRs in C57BL/6J mice. Motif density: The plot of cumulative motif density for all CEBPA, HNF4A, and FOXA1 TFBRs -/+ 1,000bp from the TFBR summit shows a distinct increase of motif density within ∼300bp around the summit for each TF (coded by color, as per Figure 1). This is in contrast to the density of each motif across 50,000 random regions (gray). Motif occurrence: In the 300bp region of high motif density around the TFBR summit, the motif occurrence is clearly higher for TFBRs compared to random 300bp region (pie charts). On average we can find motif in 99.1%, 89.2%, and 98.4% CEBPA, HNF4A, and FOXA1 TFBRs, respectively. De novo motif: We can find motif de novo in the 2,000 highest and lowest intensity TFBRs (+/−25bp around the summit), unlike for random genomic regions (data not shown). (E) When considering shadow regions (e.g., regions that are unbound in an anchor species but where in orthologous regions in different species TFs are bound), the overlap of TFBRs between pair of species anchored on C57BL/6J does not dramatically change with increasing leniency of peak calling, and closely follows the calculated rate of TFBRs divergence during evolution obtained using standard peak calling parameters, as per Figure 1B.

We performed our experiments using tissues from rodents at evolutionary distances ranging from 1 million to 20 million years (Figure 1). The inbred species we used were from mammalian genus Mus (Figure S1A available online), namely laboratory strains C57BL/6J and A/J (mostly *Mus musculus domesticus* [Mmd] [Yang et al., 2011]), wild-derived CAST/EiJ (mostly subspecies *Mus musculus castaneus*, separated from Mmd by 1 million years [MY]), as well as two more distant species—SPRET/EiJ (*Mus spretus*, separated by 3 MY) and Caroli/EiJ (*Mus caroli*, separated by ∼4–6 MY), with *Rattus norvegicus* (separated by 15–20 MY) as an outgroup. The genomes of four of these mouse species were recently reported (Keane et al., 2011), and the genome of Caroli/EiJ was sequenced specifically for this study (D.T., J.C.M., A.B., D.J.A., and P.F., unpublished data). Although the mice in this study are a combination of strains, subspecies, and species, for the sake of simplicity, we refer to all as different mouse species.

Exploiting multiple species of inbred mice unlocked a number of powerful analytical approaches to explore the quantitative and qualitative changes occurring in TF binding evolution. Relative to the reference mouse genome, our study’s mouse species have had few large-scale genome rearrangements, simplifying the identification of orthologous TF binding (Keane et al., 2011). Sequence changes between mouse species were sufficiently modest to assign a specific site of genetic variation to a corresponding TF binding location, often unambiguously. Each species has a different but well-characterized evolutionary distance from the reference C57BL/6J, which enabled analyses demanding the reliable reconstruction of ancestral regulatory states.

We determined the genome-wide binding in livers of five mouse species and rat for CEBPA, HNF4A, and FOXA1 by performing chromatin immunoprecipitation (ChIP) experiments coupled to high-throughput sequencing in biological duplicates (Figures 1 and S1A). We analyzed ChIP experiments using a native genome for each species (e.g., SPRET/EiJ ChIP experiments were analyzed against the SPRET/EiJ genome). These TFs were selected, in part, because they are representative TFs that evolve and function similar to other tissue-specific regulators in mammals (Kunarso et al., 2010; Schmidt et al., 2010). The amino acid sequences of the three TFs are highly conserved; few changes occurred between mouse species, and none were in DNA binding domains or antibody recognition sites. We defined transcription-factor-bound regions (TFBRs) as those called in both individual biological replicates and in the pooled sample; this definition removed the very lowest intensity and sporadic TF binding sites (Figure S1B). These TFBRs were the basis of all further analysis, except when clearly indicated in direct comparison of single replicates. We found similar numbers of TFBRs in all four species of mice (on average, ∼46,000 TFBRs for CEBPA, 60,000 for HNF4A, and 55,000 for FOXA1, SD between 6,200 and 10,900; Figure S1C). Although our data showed that the total number of TFBRs changes little between these closely related species, Caroli/EiJ was found to have overall fewer bound locations, most likely due to differences in the genome qualities (Figure S1C).

For each data set, we estimated our false positive rate to be less than 1% by comparing our ChIP experiments to either a mock ChIP lacking the specific antibody or input DNA from the livers; this false positive rate is similar to prior studies (ENCODE, 2012; Pickrell et al., 2011). TFBRs were found to almost always center on a sequence match for the known TF binding motif (Figure S1D); similarly, computational analyses of the sets of TFBRs with either highest or lowest ChIP intensities readily produced the known position weight matrix (PWM) when subjected to de novo motif discovery (Figure S1D). Although some fraction of TF binding likely captures indirect interactions, the high occurrence of motifs at peak summits, even in the least intense ChIP enrichment, is consistent with a substantial fraction of identified TFBRs representing direct protein-DNA contacts. Furthermore, prior studies have validated that a TF’s direct DNA occupancy at specific genomic sites is accurately captured by the in vivo crosslinking that precedes ChIP experiments (Kaplan et al., 2011; MacArthur et al., 2009). For additional methodological details, please see the section “Determination of TF-Bound Regions in Five Closely Related Mammals” in the Extended Experimental Procedures.

In sum, our experiments identified reproducible, genome-wide binding data for three liver-specific TFs with highly conserved protein sequences and cellular functions in matched tissues from five mouse species.

### The Accumulation of Differences in TF Binding in Different Mouse Species Corresponds with Interspecies Evolutionary Distance

We first assessed how rapidly TF binding differences accumulate among these five mouse genomes by determining the proportion of HNF4A, CEBPA, and FOXA1 TFBRs that reciprocally overlap between species in a qualitative manner; that is, how often TF binding in one species was evaluated as not identified in the homologous position in a second mouse species when comparing present-absent binding calls. This qualitative evaluation categorized TFBRs as either shared or unshared in a particular pair of species; the choice of binding cutoff and effect of varying this cutoff is explored in Figure S1E.

**Figure S2:**
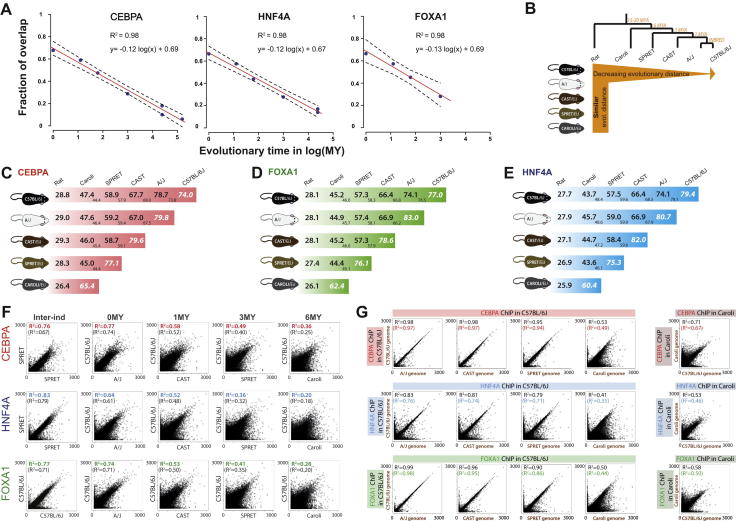
TF Binding Divergence across Closely Related Mouse Species, Related to Figure 1 (A) The rate of decay of TF binding overlap over 20-180 million years (MY) of evolution is linear when plotted as log of MY. C57BL/6J is used as a reference for calculating the overlap and evolutionary distance. In addition to our data spanning 6-20 MY, the comparison over 80-180 MY years additionally used data from Schmidt et al. (2010). Namely, for CEBPA we plotted the dog (80 MY of divergence), the human (80 MY of divergence) and the opossum (180 MY of divergence). For HNF4A we plotted only the overlap between C57BL/6J and dog and human. No additional data were available for FOXA1. The mouse strain A/J has been removed due to the incompatibility of the evolutionary distance from C57BL/6J (0 MY) and the logarithmic scale. (B) The fraction of overlapping TFBRs between five mouse species and a rat shows in vertical direction the overlap between similarly distant species (e.g., Rat versus C57BL/6J, A/J, CAST/EiJ, SPRET/EiJ, Caroli/EiJ in the first row) while in horizontal direction it follows the decreasing evolutionary distance. (C–E) The percentage of overlapping TFBRs between each pair of mouse species and rat for FOXA1 (D), HNF4A (E), and CEBPA (C) is robust to our choice of anchor species (the values in vertical line of the matrix are within ± 2 standard deviations). We see similar overlap if we consider only genomic regions that align between C57BL/6J and rat (the small print number at the bottom right of each overlap percentage), showing that the changes in TF binding are not solely accumulating in fast evolving Mus genomic regions. Far-right diagonal overlap in italics shows the proportion of TFBRs that overlap between two replicates from two individuals of the same species. (F) The correspondence of TFBR intensities between individuals is shown quantitatively by correlating two Inter-pools, each containing two biological replicates of SPRET/EiJ (for CEBPA, HNF4A) or C57BL/6J (for FOXA1), showing high correlation coefficients when we consider all TF binding regions (R^2^ in brackets) or only the overlapping TF binding regions (R^2^ in color, top left corner for each plot). This correlation of intensities decays with evolutionary distance, as shown by plotting the intensity for orthogonal TF binding regions in C57BL/6J and A/J, CAST/EiJ, SPRET/EiJ, and Caroli/EiJ. (G) The evolutionary decay of intensity correlation is not the result of a mapping bias. Correlation of the intensities of all TF binding regions from the same C57BL/6J ChIP-seq sample aligned either to its own genome (“C57BL/6J genome”) or to genomes of related mouse species, illustrates the most extreme effect of miscalling all SNVs on read alignment for CEBPA, HNF4A, and FOXA1. From left to right, these are all TF binding regions in C57BL/6J aligned to NCBI37 genome (its own “C57BL/6J genome”) versus A/J, CAST, SPRET and Caroli genomes. In addition, Caroli ChIP-seq sample is also aligned to Caroli versus C57BL/6J genome (far right). The R^2^ is based on Pearson correlation for overlapping and all TF binding regions (black and in color + brackets, respectively) and is listed in top left corner.

Qualitative differences in mammalian TF binding, even in short evolutionary distances, appear to accumulate at an exponential rate of e^−0.12∗(Million Years)^ (Figures 1B, S2A, and S2B). Because this rate is higher than that observed for *Drosophila* species, estimated to be at considerably greater evolutionary distances (Bradley et al., 2010; He et al., 2011b), we attempted to control for as many nonbiological sources of variation as possible. We first confirmed that the addition of ChIP data from humans and dogs did not alter this decay rate (Figure S2). We then established that our calculation was robust to (1) the choice of anchor species for the analysis (Figures S2B–S2E); (2) whether we consider the entire mouse genome or only those regions alignable with rat, which controls for the potential effect of *Mus* lineage-specific large indels on the rate of TF binding divergence (Figures S2B–S2E); and (3) the particular binding threshold chosen to define TFBRs (Figure S1E). For (3), we analyzed whether using a threshold during our peak calling for TFBR, which removed lowest-intensity peaks, caused us to overestimate the rate at which TF binding differences accumulate between species. We took the complete set of TFBRs in all five species and identified the orthologous aligned regions that were called as unbound in any mouse lineage or lineages. Specifically within this set of orthologous unbound regions, we systematically recalculated the rate at which differences accumulate by increasing the leniency of the peak-calling threshold (Figure S1E). Regardless of the threshold used, TF binding differences always appeared to accumulate at rates near to e^−0.12∗(Million Years)^.

We sought to establish whether homologous TF binding sites showed quantitative differences in their genomic occupancy between any two mouse species, similar to that observed among fruit fly species (Bradley et al., 2010). Similar mechanisms have been suggested to contribute toward interindividual variability in genetically heterogeneous humans (Kasowski et al., 2010; McDaniell et al., 2010). We first compared how replicate binding experiments for the same TF differ among distinct C57BL/6J individuals by plotting ChIP intensities against each other in an X-Y scatterplot. Both on a site-specific and genome-wide basis, TF binding profiles of different individuals with the same genetic background were highly similar (Figures 1 and S2F). Comparison of the individual replicates and combinations of these replicates for our three TFs showed interindividual correlations ranging between R^2^ = 0.76 and 0.83. This baseline correspondence between ChIP-seq experiments performed in different but genetically identical mice shows the expected total quantitative variation caused by the combination of biological variation in TF binding between individuals and technical aspects of the ChIP protocol.

We then performed similar analysis for the shared TFBRs defined above to establish how rapidly TFBR intensities diverge between different mouse species. This revealed greater variability between any two mouse species than within one species in the relative TF binding intensities; importantly, this variability increased in correspondence with evolutionary distance (Figures 1 and S2F). We considered the possibility that inaccuracies in our assembly of the underlying mouse genomes may contribute to the observed TF binding differences by estimating the maximum possible contribution this could make to our data. We mapped the C57BL/6J sequencing reads from ChIP experiments onto the genomes of each of the other species and then inspected the resulting loss of correlation. Little difference was observed except in the case of the most divergent species Caroli/EiJ (Figure S2G) and, in all cases, the differences were less pronounced than the observed loss of intensity correlation in our experiments. For additional methodological details, please see the section “The Accumulation of Difference in TF Binding in Different Mouse Species Corresponds with Interspecies Evolutionary Distance” in the Extended Experimental Procedures.

In sum, the qualitative differences (i.e., fraction of unshared TF binding) between closely related mouse species appear to accumulate considerably more quickly than was found in highly divergent *Drosophila* species (He et al., 2011b), which are thought to be at a chicken-human distance (Lin et al., 2008). In mammals, both the location and the intensity of TF binding differ rapidly with the increasing evolutionary distance.

### Variations in Bound Genetic Sequences Can Account for Only a Fraction of TF Binding Differences among Closely Related Mammals

**Figure 2:**
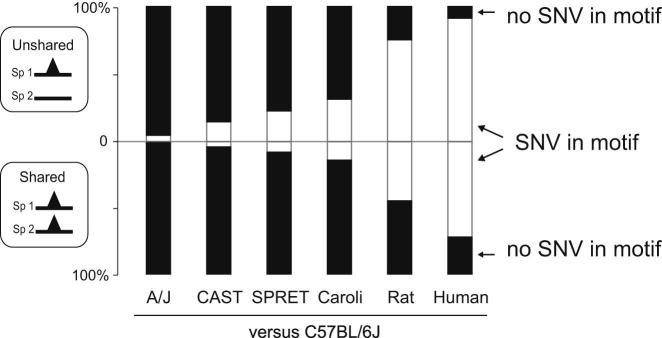
Evolutionary Differences in TF Binding Cannot Be Explained Purely by Genetic Variation in Directly Bound Sequence Motifs We categorized CEBPA binding events by whether they were unshared (top bar chart) or shared (bottom bar chart) between C57BL/6J and a second species. We then identified whether the directly bound motif is identical (black shaded) or contains genetic variation (white shaded). Variation increased with evolutionary distance; unshared binding events had SNVs in their bound genetic sequences at a slightly higher frequency (p < 2.2 × 10^−16^ with Fisher’s exact test). The vast majority of peaks do not have genetic sequence variations within their directly bound motifs; importantly, this is true for unshared peaks where, for instance, less than a quarter of C57BL/6J peaks not found in SPRET/EiJ have variation from the C57BL/6J reference. See also Figure S3.

We sought to estimate the maximal extent to which SNVs between mammalian species could be directly responsible for the qualitative differences in TF binding. We additionally reanalyzed published ChIP-seq data for HNF4A and CEBPA in human, dog, and opossum to capture more distant evolutionary outgroups (Schmidt et al., 2010). Analyzing each species pair separately, we categorized the TF binding in C57BL/6J by whether it was present in an orthologous location in the second species (Figure 2, left-hand *y* axis). For the shared and unshared TF binding, we then identified the sequences matching the TF’s known binding motif nearest to the TF binding maximum in C57BL/6J and asked whether these motifs contained an SNV in the second species (Figure 2, right-hand *y* axis).

**Figure S3:**
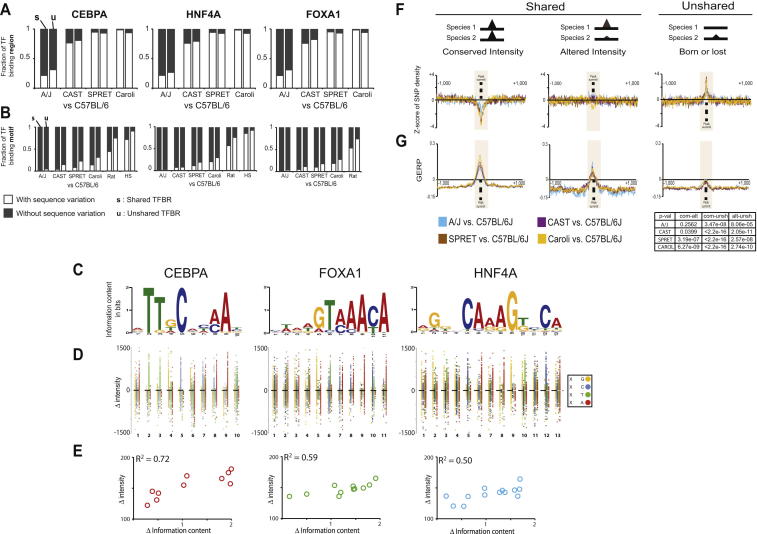
Correlation of TF Binding and Its Underlying Sequence Variation, Related to Figure 2 (A) Pairwise comparison of C57BL/6J and each other species identified the shared and unshared (respectively, s and u) TF bindings regions. Within each category, the TFBRs with sequence variation (in white) and without sequence variation (in black) within +/−150 nt around the peak summit are shown. (B) In pairwise comparisons as per (A), the TFBRs with sequence variation (in white) and without sequence variation (in black) in the central canonical motif for each TF are shown. (C) The logo representation of the position weighted matrix for each TF’s motif. (D) For CEBPA, FOXA1, and HNF4A, TF binding sites that had a SNV in the directly bound motif were collected. Each of these SNVs was plotted based on its location in the canonical motif (*x* axis), and the TF binding intensity difference with which it was associated (*y* axis). Each point was color-coded by the base occurring in C57BL/6J; for instance, a T in C57BL/6J is plotted as green (where in another species, the same base is A, G, or C), A in red, G in orange and C in blue. (E) The difference in TF binding intensity is correlated with the change in information content of the TF binding motif of CEBPA, HNF4A and FOXA1. (F) The distribution of Z-score of single nucleotide variation (SNV) density in a region of +/−1000 bp around the CEBPA summit is shown for the five mouse species. Compared to the background genome, the SNV densities between the genomes of C57BL/6J and A/J, CAST/EiJ, SPRET/EiJ or Caroli/EiJ are strongly depleted in the TF binding regions with conserved intensity, but similar for TF binding that varies in intensity, and strongly enriched for unshared TF binding. (G) The distribution of GERP scores (Genomic Evolutionary Rate Profiling) in a region of +/−1000 bp around CEBPA summits is shown for the five mouse species. Using this criteria, the TF binding regions with conserved intensity show much greater sequence constraint than regions with altered intensity or where TF binding was present in one species but not in another by pairwise comparison. The t –test based p-value for each comparison and species is listed on the right.

The resulting plot revealed that, as expected, the frequency of motifs with SNVs increases steadily with increasing evolutionary distance from C57BL/6J in both shared and unshared TFBRs; somewhat unexpectedly, in every mouse species, the large majority of both shared and unshared TFBRs are bound to genetic sequences with no sequence variations in their motifs. Across the *Mus* genus, SNVs in directly bound sequences matching the canonical motif could account for less than a third of TF binding differences between species; the overall result was largely independent of the information content of the base where SNVs occurred (data not shown). For instance, the maximum fraction of the changes in TF binding between C57BL/6J and Caroli/EiJ that might be assigned purely to genetic changes in the bound motif was typically near a quarter of the total (31.2% [CEBPA], 29.6% [HNF4A], and 27.5% [FOXA1]). Typically, a sixth of the peaks shared between C57BL/6J and Caroli/EiJ have an SNV in the directly bound motif (14.1% [CEBPA], 20.9% [HNF4A], and 18.6% [FOXA1]) (Figures S3A and S3B), which is consistent with recent reports (Kasowski et al., 2010; Reddy et al., 2012). Thus, differences in genetic sequences can be the primary determinant only for a modest fraction (typically 10%–20%) of TF binding differences between these mammalian genomes.

We searched for the exact types of sequence variations associated with altered TF binding that were more likely to be causal. By mapping the specific variants associated with either increased or decreased intensity of TF binding between species, we discovered that, in the minority of cases in which SNVs were associated with TF binding differences, the base variations that introduced preferred high-information content bases within the motif tended to increase the strength of associated TF binding. Our results therefore support prior reports that motif positions with high information content can be more important for TF binding (Figures S3C–S3E) (Reddy et al., 2012; Schmidt et al., 2012; Spivakov et al., 2012).

Still, the large majority of TF binding differences are not associated with genetic changes during evolution to the directly bound sequence motifs, and shared TF binding peaks with conserved intensity (discussed below) were more likely to show depletion of nucleotide substitutions (Figure S3F) and heightened sequence constraint (Figure S3G). For additional methodological details, please see the section “Variations in Bound Genetic Sequences Can Account for Only a Fraction of TF Binding Differences among Closely Related Mammals” in the Extended Experimental Procedures.

In sum, TF binding can be conserved where directly bound genomic motifs differ; on the other hand, the large majority of changes in TF binding among closely related species are not associated with changes in the observed motifs. This complex relationship between differences in TF binding and differences in underlying genetic sequences between closely related mammals is similar to prior reports in more divergent *Drosophila* species (Biggin, 2011; Bradley et al., 2010; He et al., 2011b).

### TF Binding in Combinatorial Clusters Evolves Coordinately

**Figure S4:**
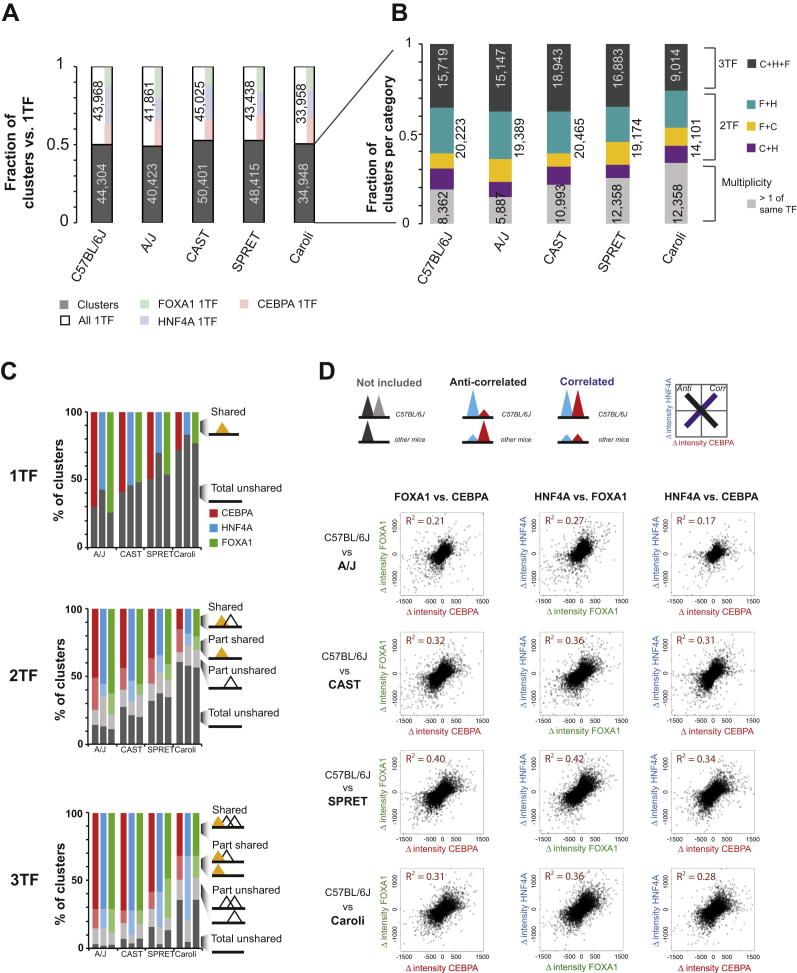
Coevolution of TF Binding Occurring in Clusters, Related to Figures 3 and 4 (A) Number of TFBRs with a single TF binding (1TF) or clusters of multiple TF binding. The ratio of clusters (gray) to 1TF (white) occurrences is similar in each mouse species. Within singletons, the proportion of free-standing CEBPA, HNF4A, and FOXA1 is shown by color as an inset (right side of each white bar). (B) Number of each sub-classification of the clusters of co-bound TFs. The largest fraction are generally the 3TF clusters, containing precisely one of each TF (gray, inset is the total number of TFBR). Within the 2TF clusters (multicolored, right outset is the total numbers of TFBR), the 2TF clusters containing FOXA1 and HNF4A (F+H, blue) are more often found than the combinations of the other two TFs (F+C, yellow; C+H, purple). A minority of clusters has multiple TFBRs for the same factor(s) (light gray inset is total number of TFBR). (C) TF binding events occurring in relative isolation (1TF) are far more likely to vary between species than binding events found in a cluster of TF binding with two TFs (2TF) or three TFs (3TF). When analyzed from the point of view of a specific TF (in yellow on right annotations), we define part shared as TFBR where co-bound TFs are missing in a second species, part unshared as those where co-bound TFs are present but the anchor TF is absent, and total unshared as TFBR lacking any TF occupancy in a second species. (D) The intensity differences between species of TFs co-bound in clusters differ in a coherent manner. All regions that were co-bound by two or more TFs and shared between C57BL/6J and any second mouse species (A/J, CAST/EiJ, SPRET/EiJ or Caroli/EiJ) were interrogated to see if, on average, we detect correlated or anti-correlated evolution of TF binding intensities. Each scatterplot shows the inter-species difference in intensity for one TF versus a second TF; for instance, in the first column, we plot the difference in intensity of FOXA1 versus the difference in intensity of CEBPA for each species-pair. The changes in TF binding intensities are consistently correlated. Pairs of TFs that were only partially conserved or differ entirely in occupancy between the C57BL/6J and other species were not considered.

Because few differences in TF binding between mouse species could be connected to specific SNVs in the motif, we explored whether the extent of combinatorial binding among CEBPA, FOXA1, and HNF4A could help to explain these differences. Within each species, we first identified the singleton 1TF positions where a binding event for any one of the TFs in this study occurred in complete isolation. We then categorized the remaining regions with overlapping binding of HNF4A, CEBPA, and/or FOXA1 as clusters of TF cobinding. We defined 2TF and 3TF binding clusters as locations bound by two or three TFs within a 300 bp window with strictly singular TF binding (e.g., a 3TF cluster has exactly one TFBR for each component factor). The 1TF, 2TF, and 3TF categories captured the large majority of TF binding events (Figures S4A and S4B). The remaining TFBRs were assigned to a category containing regions of binding multiplicity representing locations in which the same TF binds repeatedly in close proximity. Our categorization of the C57BL/6J binding data was typical—1TF singletons represented 49% of the regions bound in the genome, 2TFs were 23%, 3TFs were 18%, and multiplicity locations were 9%; other species of mouse showed similar distributions. For full methodological details, please see the section “TF Binding in Combinatorial Clusters Evolves Coordinately” in the Extended Experimental Procedures.

**Figure 3:**
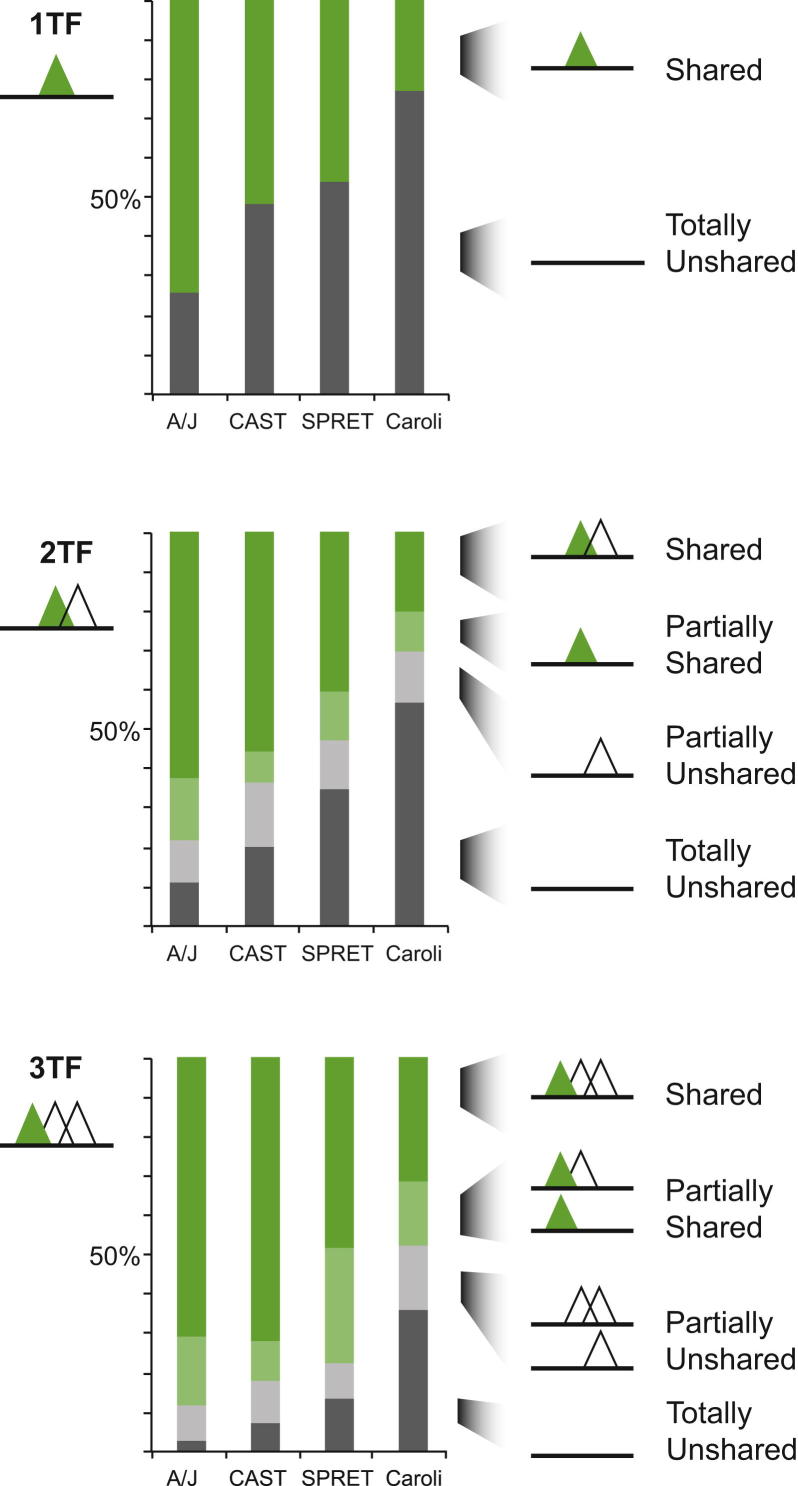
Regions Bound by Multiple TFs in C57BL/6J Are More Likely to Be Found in a Second Mouse Species The probability that FOXA1 (and/or its partners) will be lost depends on the TF binding neighborhood. FOXA1 binding occurring in isolation (1TF) is far more likely to be lost than binding events found in a TF binding cluster with two TFs (2TF) or three TFs (3TF); these cases represent loss of all factors simultaneously (labeled as totally unshared). See also Figure S4 for similar plots for CEBPA and HNF4A.

We discovered that the more mammalian TFs were present in a cluster, the less likely a component TF binding site was to be entirely lost between species (Figures 3 and S4C). For instance, the fraction of FOXA1 binding regions shared between C57BL/6J and A/J steadily increased from 73.4% (1TF) to 77.0% (2TF) to 88.5% (3TF). Indeed, isolated TF binding appears to be relatively unstable; fully a quarter of 1TF sites vary between the closely related strains C57BL/6J and A/J (Figure S4C). It is important to note that our cluster categorization is limited by the fact that it uses only a modest subset of the liver-specific TFs known to control tissue-specific gene expression (Odom et al., 2006); inclusion of more TFs may reveal that regions with higher combinatorial binding (e.g., 4TF and 5TF clusters) would be even more often shared among different mouse species.

In summary, increasing the number of TFs within a specific genetic locus greatly increased the probability that component TF binding would be shared between closely related mammals.

### TF Binding Intensities within Clusters Coevolve

We further considered the possibility that TF binding intensities are coevolving, as has been observed for *Drosophila* (Bradley et al., 2010). Coevolution in this case means that, if the TF binding intensity of a component TF within a cluster differs between two mouse species, then the intensities of cobound TFs are more likely to differ as well and in a coherent direction. For instance, suppose there was a region directly bound by both HNF4A-FOXA1 in C57BL/6J where the homologous FOXA1 binding in SPRET/EiJ had greater binding intensity—would HNF4A intensity also be greater?

**Figure 4:**
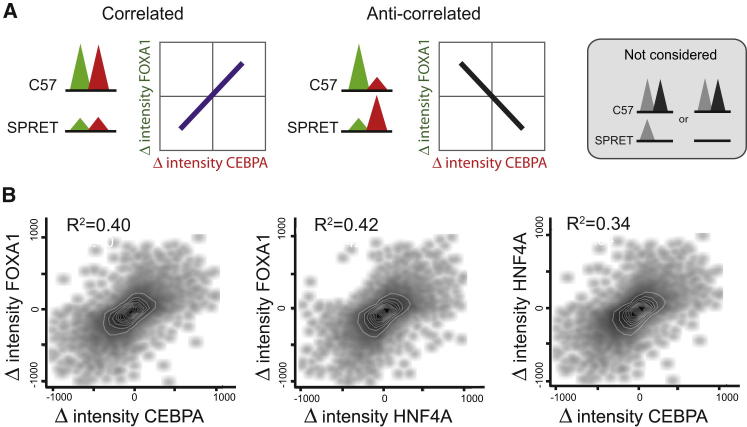
TF Binding Intensities Differ in a Positively Correlated Manner (A) For each pair of TFs, all regions that were cobound and shared between C57BL/6J and SPRET/EiJ were identified. Each scatterplot shows the change in intensity for one TF versus the second TF between these species. (B) Combinatorial TF binding intensities coevolve. The differences in TF binding intensities showed good correlation between different TFs, suggesting coordinate evolution. Comparisons of C57BL/6J with each of the other mouse species show similar results. See also Figure S4.

Within the 2TF and 3TF clusters, we identified pairs of TFs whose binding was shared between two mouse species and then plotted the change in binding intensity of each TF against the other (as shown for C57BL/6J and SPRET/EiJ in Figure 4; see also Figure S4D and the section “TF Binding Intensities within Clusters Coevolve” in the Extended Experimental Procedures). We consistently found positive correlations between all pairs of TFs (typical values R^2^ = 0.4). This result is consistent with a model in which indirect influences, such as changes in the local chromatin environment (John et al., 2011; Li et al., 2011), additional coacting transcriptional regulators (Biddie et al., 2011; Harrison et al., 2011; Nien et al., 2011), and/or indirect cooperativity among cobound TFs (Mirny, 2010) have substantial influence on levels of combinatorial TF binding.

Thus, in clusters of combinatorial TF binding, differences in binding intensities between species appear to occur coordinately, and the component HNF4A, CEBPA, and FOXA1 binding sites increase and decrease their genomic binding strengths in a coherent, directional manner.

### A Large Core Set of TF Binding Intensities Is Evolutionarily Stable across All Five Mouse Species but Is Decoupled from Functional Target Genes

We then asked whether TF binding intensity also correlated with the probability that TF binding was shared in closely related mammals. Results from prior studies in mammals (Kunarso et al., 2010; Schmidt et al., 2010) and *Drosophila* (Bradley et al., 2010; He et al., 2011b) have appeared contradictory. In mammals, there appears to be minimal correlation, if any, between TF binding intensity and their presence at orthologous regions in divergent vertebrate species; however, in flies, TF binding intensity and TF binding conservation appear to correspond closely.

**Figure 5:**
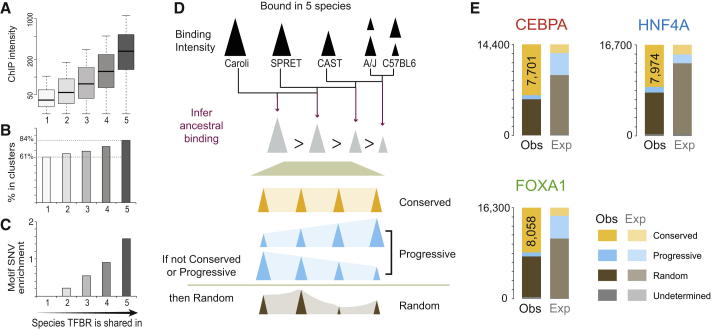
TF Binding Regions Shared in All Five Mice Often Show Higher and More Constrained Binding Intensity (A) The average intensity of TF binding increases with the number of species in which FOXA1 is bound. (B) FOXA1 binding regions shared among more species are more likely to be part of combinatorially bound regions. (C) The FOXA1 binding regions shared among more species are more likely to be robust to SNVs in the underlying FOXA1 motif. (D) The ancestral intensity for each TF binding region in the mouse genome at four ancestral nodes was inferred using parsimony and was used to establish whether TF binding intensity was conserved or monotonic during evolution. If neither model was matched, then binding regions were classified as evolving randomly. (E) The expected distribution of conserved, directionally changing, and randomly changing TF binding intensity over time was established by randomizing the intensities of each species’ bound regions. A few TFBRs fell outside this classification due to occasions of inherent ambiguity in inferring ancestral binding intensity; these are listed as undetermined. In comparison, observed in vivo TF binding data consistently showed depletion of directed evolution and strong enrichment of conserved binding intensities (p < 10^−6^). See also Figure S5.

**Figure S5:**
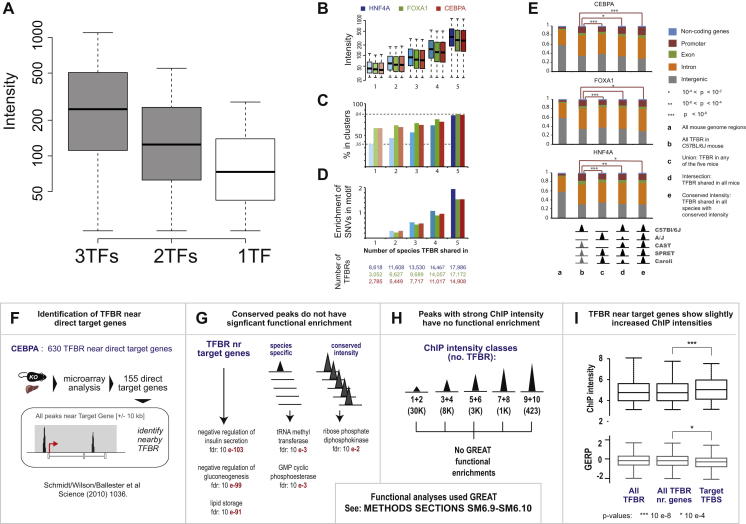
Relationships among TF Binding Intensity, Cluster, Conservation, and Function, Related to Figure 5 (A) The distribution of ChIP intensities, plotted by number of nearby TFs. 3TF-clusters and 2TF-clusters are shown in gray, and singly-bound 1TF sites are shown in white. On average, the more TFs are present in a cluster, the stronger the ChIP enrichment. (B) The average intensity of TF binding increases in line with the number of species TFBRs are found in. (C) TFBRs shared among more species are more likely to be part of combinatorially bound clusters. (D) The TF binding regions shared among more species are more likely to be robust to SNVs in the underlying TF motif. (E) The set of TFBR shared by all five mouse species with conserved intensity (column e) and without conserved intensity (column d) are slightly (but statistically significantly) more likely to occur in promoter regions, when compared to the whole set of C57BL/6J TF binding regions (column b). (F) 155 direct target genes of CEBPA were selected by identifying, with microarray analysis, the genes with a significant expression decreases in a CEBPA KO mouse liver, when compared to WT liver (see Schmidt et al., 2010). The TFBRs located in a region from −10 Kb from the target gene 5′ start to +10 kb from the target gene 3′ end were selected (in further panels: TFBR near target gene). (G) When compared to TFBR near target genes, both species-specific TFBRs and TFBRs shared by all five species with a conserved intensity show very weak functional enrichment. This analysis was performed using the GREAT tool, and representative classes of highest statistical enrichments are shown for all TFBR categories. (H) We sorted TFBR in C57BL/6J mice by binding intensity into ten classes, and our search using the GREAT tool for evidence of functional enrichment was unsuccessful. (I) The TFBR near target genes shows a slight increase of ChIP strength compared to the TFBR near all genes. The conservation across the 29 mammals of the underlying sequence is only marginally significantly higher in this category.

We therefore categorized TFBRs based on how many mouse species they occurred in and discovered that, within one mammalian genus, there are steadily increasing intensities for each TF with increasing depth of TF binding conservation (Figures 5 and S5). Regions containing a deeply shared TF binding site were also more likely to have combinatorial TF binding (Figures 5B and S5C) and to be tolerant of genetic variations within bound motifs (Figures 5C and S5D). Together, our data indicate that a large set of highly conserved, combinatorial, and intense binding regions exist in all five mouse species, showing molecular features similar to those observed in TF binding comparisons between more divergent *Drosophila* species (Bradley et al., 2010; He et al., 2011b).

We then tested three key predictions of recent models proposed for TF binding evolution and function in animals (Biggin, 2011): (1) that TF binding intensities (as opposed to the genetic sequences) of the bound regions present in all mouse species should be under strong constraint; (2) that regions bound strongly and consistently in multiple species should capture the known TF functionality; and (3) that TF binding near functional target genes should be of stronger intensity.

To test the first hypothesis, for each TF, we analyzed all five species’ worth of ChIP data to identify a set of ∼14,000 binding events bound across all mouse species and inferred the TF binding intensity profiles of four common ancestors using Wagner parsimony (Figure 5D). Subsequently, we classified each TFBR into one of three categories: (1) conserved intensity, similar intensities across all ancestral states; (2) progressively changing intensity, the intensity of successive ancestral TFBRs progressively increases or decreases; (3) randomly changing intensity, when a locus has neither a conserved nor progressive profile.

As a control, we repeated this analysis after reassigning the TFBR intensities randomly within each species to different loci, which generated a background expectation that assumes random divergence.

For the three TFs in our study, approximately half (47%–56%) of all TFBRs have conserved intensities, somewhat fewer of them (40%–46%) are random, and a small percentage (4.0%–6.4%) are progressive. When compared with the randomized expected background, these distributions reveal strong enrichment toward conservation at the expense of both progressive and stochastic evolution (p < 10^−6^) (Figure 5). This result is robust to the definition of intensity classes, the definition of similarity, and the inclusion or exclusion of missing binding events.

We then asked whether conserved binding in multiple mouse species could predict functionality. We first identified the TFBRs located near genes whose transcription is altered by CEBPA knockout in a genetically engineered mouse (Hatzis et al., 2006; Schmidt et al., 2010) and then used the GREAT algorithm (McLean et al., 2010) to compare the functional enrichments of specific TFBRs relative to the entire set of TFBRs in C57BL/6J. As expected, these positive-control TFBRs showed extremely significant liver-related functional enrichments (Figures S5F and S5G). The conserved intensity peaks showed no obvious enrichment for liver-related functions. By sorting TFBRs into ten intensity classes and analyzing their functional enrichments, we further established that TFBRs with the strongest TF binding intensity do not occur preferentially near genes systematically enriched for any biological function (Figure S5H). Therefore, our data indicate that neither TFBRs with constrained binding intensity nor those of stronger genomic occupancy reveal functionally enriched regions; this result appears to differ substantially from related findings in *Drosophila* (Biggin, 2011).

Third, we established that the peaks occurring near genes transcriptionally dependent on CEBPA, which were identified using the knockout mouse, had slightly stronger ChIP enrichments when compared with all TFBRs (p < 10^−8^) (Figure S5I). Similar analyses using direct targets of HNF4A (Boj et al., 2009) to explore TF function and TF binding intensity afforded similar results (data not shown). For additional methodological details, please see the section “A Large Core Set of TF Binding Intensities Is Evolutionarily Stable across All Five Mouse Species but Is Decoupled from Functional Target Genes” in the Extended Experimental Procedures.

In summary, regions with stronger TF binding intensities involved more TFs and were less likely to be lost over evolutionary time. Within the conserved TF binding regions shared among all five mouse species, we observed more than 7,000 loci where the TF binding strength is constrained, and these loci, perhaps surprisingly, do not appear to be concentrated near functional target genes.

### The Genetic Deletion of a Single TF Has a Direct Effect on the Stability of the Remaining TFs within a Cobound Cluster

**Figure 6:**
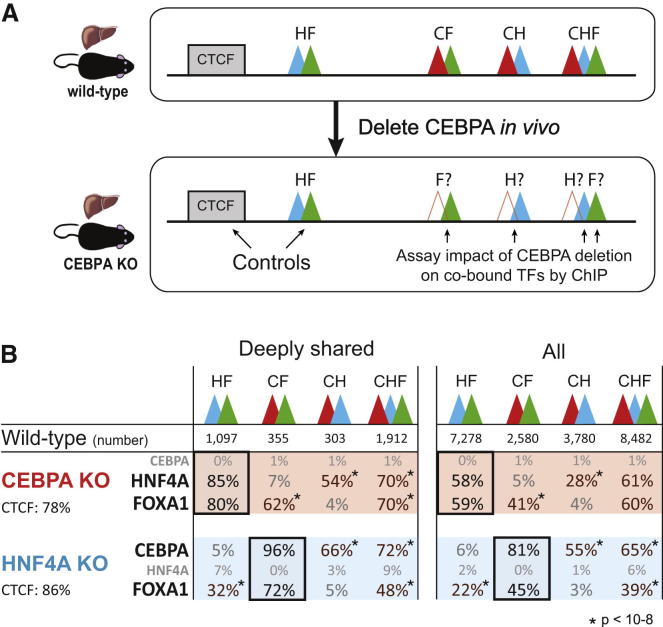
Effect of Knocking Out CEBPA and HNF4A In Vivo on the Binding of the Remaining TFs in Cobound Clusters (A) Livers from a genetically engineered CEBPA KO mice were obtained, and ChIP experiments were performed against HNF4A and FOXA1 to evaluate the impact of CEBPA deletion on cobound TFs located in clusters ([HNF4A (H, blue), FOXA1 (F, green), and CEBPA (C, red)] and CTCF [a noninteracting partner, as a control]). Corresponding experiments were performed using HNF4A KO mice. (B) The effect of knocking out CEBPA and HNF4A was evaluated for the following cluster classes: 2TF clusters containing HNF4A and FOXA1 (HF), CEBPA and FOXA1 (CF), and CEBPA and HNF4A (CH) and finally, all three TFs (CHF). Two TF binding experiments served as controls in CEBPA and HNF4A KO mice: CTCF, which binds independently of tissue-specific TFs (Faure et al., 2012), and the 2TF clusters not containing the deleted factor (in darker black frames) because knocking out CEBPA should not affect the binding of HNF4A or FOXA1 found in HF clusters, and knocking out HNF4A should not affect CF clusters. The TF binding differences in WT versus KO show that genetically removing one of the TF found in a cluster destablizes the binding of cobound TFs. This effect is in almost all cases statistically significant and is observed for both deeply shared (left) and all (right) 2TF and 3TF clusters. See also Figure S6.

We asked what effect genetic deletion of single component TFs would have on the stability of combinatorial TF binding and how the genetic stability is related to the evolutionary conservation of the TF binding within these clusters. We obtained livers from genetically engineered mice lacking either HNF4A or CEBPA. Although we cannot entirely rule out the influence of indirect effects, each TF knockout had minimal effect on the gene expression of the other liver-specific TFs (Kyrmizi et al., 2006; data not shown). We then performed ChIP-seq experiments against HNF4A, CEBPA, and FOXA1. These experiments further confirmed that both genetic knockouts were successful and that the targeted TF was largely absent from liver (Figure 6).

We then asked what effect these genetic deletions have on 2TF and 3TF clusters that were consistently bound across all species of mice, expecting that these would be most robust to perturbations. We used two internal controls that should be unaffected by the deletion of a specific TF: (1) CTCF binding, which occurs in the genome independently of tissue-specific TF clusters (Faure et al., 2012); and (2) the 2TF clusters not containing the deleted factor (Figure 6A). Our data confirmed that CTCF binding was unperturbed by knockout of the unrelated factor, as was TF binding in the 2TF clusters lacking the deleted regulator. The use of multiple internal controls afforded robustness to our analysis.

**Figure S6:**
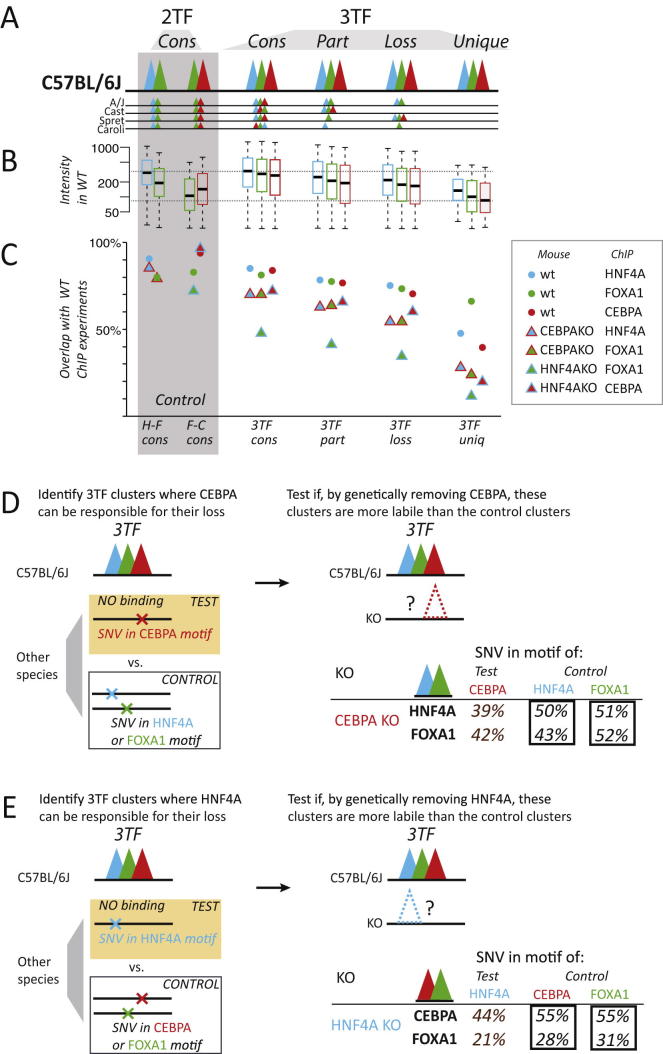
Genetic Knockout of CEBPA and HNF4A to Destabilize Combinatorially Bound Regions, Related to Figure 6 (A) 3TF binding in C57BL/6J was sorted by how deeply conserved these 3TF clusters were as follows: *Cons*, all five species; *Part*, partially bound, no total losses; *Loss*, total loss in at least one other species; *Unique*, No binding at all found in any other mouse species. Clusters of 2TF found in the WT C57BL/6J and all the other mouse species are included as controls. (B) The distribution of binding intensities for each TF category in C57BL/6J are shown, color-coded (blue is HNF4A, green is FOXA1, red is CEBPA). (C) The overlap of a newly-created set of TF binding using new biological replicates in WT mouse liver were calculated versus the TFBR used throughout the manuscript (solid circles); in comparison, ChIP experiments were also performed in genetically engineered mice lacking either HNF4A or CEBPA (color-coded triangles). (D) We identified a subset of 3TF regions (1,173) in C57BL/6J where the whole cluster is absent from any of the other mouse species and where this disappearance can be linked to sequence changes in CEBPA motif only. We tested if these regions are more likely to be lost in CEBPA KO mouse liver, when compared to control regions where cluster disappearance is associated with changes in the HNF4A motif (1,882) or the FOXA1 (1,390) motif. We found that the 3TF sites identified as susceptible to CEBPA loss during evolution were also more sensitive to the genetic deletion of CEBPA. (E) We identified a subset of 3TF regions (1,882) in C57BL/6J where the whole cluster is absent from any of the other mouse species and where this disappearance can be linked to sequence changes in HNF4A motif only. We tested if these regions are more likely to be lost in HNF4A KO mouse liver, when compared to control regions where cluster disappearance can be linked to changes in the CEBPA (1,173) motif or the FOXA1 (1,390) motif. We found that the 3TF sites identified as susceptible to HNF4A loss during evolution were also more sensitive to the genetic deletion of HNF4A.

We consistently found that deletion of HNF4A or CEBPA from a combinatorially bound region caused loss of cobound partner TFs (Figure 6). For instance, genetic deletion of HNF4A has no effect on the deeply shared CEBPA-FOXA1 2TF clusters (96% overlap with wild-type [WT]) but significantly destabilizes the CEBPA-HNF4A 2TF clusters (66% overlap with WT: p < 10^−15^, Fisher’s exact test). We also observed a more modest effect on cobinding TFs within the 3TF clusters versus the 2TF clusters. The differential intensity of the different categories of TF binding could not explain the loss of TF binding observed in the knockout experiments; regardless of the details of the conservation of the 3TF clusters in WT C57BL/6J, TF binding was roughly equally likely to be lost in the knockout mouse (Figures S6A–S6C). For additional methodological details, please see the section “The Genetic Deletion of a Single TF has a Direct Effect on the Stability of the Remaining TFs within a Cobound Cluster” in the Extended Experimental Procedures.

Our multispecies TF binding data allowed us to study the effect that singular genetic mutations can have on combinatorial TF binding. We identified between 1,000 and 2,000 3TF binding clusters in C57BL/6J that were (1) absent in a second mouse species and (2) where SNVs in the second species were located in either an HNF4A or CEBPA motif. Because of the high DNA sequence identity between the strains in this study, these are locations where the absence of a single TF binding event likely resulted in absence of the entire cobound cluster. For instance, consider the set of genomic locations bound by all three factors in C57BL/6J and entirely absent in SPRET/EiJ and where an SNV was found only in an HNF4A motif; this combination of features suggests that this cobound cluster is uniquely sensitive to HNF4A binding for stability. In the CEBPA and HNF4A knockout mice (Figures S6D and S6E), we found that these 3TF sites where evolutionary analysis suggested sensitivity to loss of either HNF4A or CEBPA are also sensitive to the genetic deletion of the same factor.

In sum, the genetic deletion of a single TF has a direct effect on the stability of the remaining TFs within a cobound cluster, and this effect cannot be explained purely by differences in TF binding intensities.

## Discussion

To elucidate the first steps of TF binding evolution and the underlying mechanisms in mammals, we characterized the binding profiles of three tissue-specific TFs, CEBPA, HNF4A, and FOXA1, in livers from six inbred rodents. The recent divergence times of the selected mammals represents an optimal phylogenetic window to study the mechanisms of TF binding evolution. The evolutionary branch lengths among these five members of the *Mus* genus are an order of magnitude less than that between human and mouse, which shared a common ancestor 80 MYA. The short branch lengths between mouse species allowed us to identify how genetic variations between species contribute to the earliest interspecies differences in TF binding.

Our results demonstrate that features of tissue-specific TF binding evolution predicted from studies in other eukaryotic lineages (Biggin, 2011) also occur in mammals. First, mammals show widespread quantitative alterations in TF binding intensities, even in closely related species (as per Bradley et al., 2010). Second, although SNVs in and near directly bound motifs may be responsible for a modest fraction of these differences, other influences appear to play a larger role (Bradley et al., 2010; Kasowski et al., 2010; Reddy et al., 2012). Third, genomic regions bound by multiple regulators show coordinated alterations in their TF binding between species (Bradley et al., 2010), as during development (Li et al., 2011). Finally, when compared with isolated TF binding locations, combinatorially bound regions in mammals are more evolutionarily stable, as found for flies (He et al., 2011b). We also newly reveal that combinatorial binding is more robust to sequence variations in directly bound motifs and that the more species in which a TF binding region is found, the stronger the genomic occupancy. In short, the biochemistry and biophysics of TF binding shared among all eukaryotes dictates many common features of TF binding evolution.

The presence of more cobound TFs in a cluster corresponds with a higher probability of TF binding conservation, suggesting that a TF’s binding may influence, at least in part, the stability of cobound TFs. We functionally tested this by genetically deleting one component of the clusters and then interrogating what effect this deletion had on the stability of the cobound regulators. We found that there was a concomitant, systematic destabilization of combinatorial TF binding in the clusters containing the genetically removed TF, which was of a similar magnitude for both CEBPA and HNF4A. This general effect would be consistent with a model in which TFs compete with nucleosomes for DNA occupancy (Mirny, 2010). Similar coordinated and quantitative changes in binding being mediated via cooperativity have been identified in *Drosophila*, in which sequence changes in recognition motifs for *vfl* (*Zelda*) can explain, in part, differences in DNA binding by gap A-P TFs among closely related fruit fly species (Bradley et al., 2010).

We have discovered two striking contrasts in how TF binding evolution occurs in mammals and flies. First and most prominently, differences in TF binding locations (that is, qualitative gains and losses) accumulate between closely related mammals at an exponential rate; at 6 MY from a common ancestor, *Mus musculus domesticus* (C57BL/6J) and *Mus caroli* typically share only half of experimentally determined binding sites for these three liver master regulators. In sharp contrast, almost no variations in TF binding locations were observed between *Drosophila melanogaster* and *yakuba* (Bradley et al., 2010), which are thought to have a molecular distance greater than mouse-rat (Lin et al., 2008). Comparison of *twist* (*twi*) binding in extremely diverse fruit fly species showed that, at a molecular distance thought to be the same as chicken-human, well over half of TF binding events were found at the same homologous location in every *Drosophila* species (He et al., 2011b). Overall, despite the presence of a subpopulation of conserved TFBRs, TF binding in mammals appears to be considerably more evolutionarily labile than in flies.

Second, in flies, those genomic regions most strongly bound by a TF tend to be near the functional target genes, and this TF binding near functional target genes is present in more fruit fly species and is stronger in intensity overall (Bradley et al., 2010; Fisher et al., 2012; He et al., 2011b; MacArthur et al., 2009), which was reviewed in Biggin (2011). In our mammalian data, we observed no such clear correspondences. The TFBRs with highest genomic occupancy showed little evidence of functional enrichment relative to other TF binding events, and the well-characterized functional targets of HNF4A and CEBPA were only modestly enriched for strong TF binding. Furthermore, TF binding locations present in all five species of mice are not preferentially located near known TF target genes. Our study’s results also appear to differ from certain studies in mammals that have suggested that strength of TF binding corresponds with circadian phase-specific DNA binding (Rey et al., 2011) and possibly even dictates functionality (Rey et al., 2011; Whyte et al., 2013).

If the many molecular similarities in TF binding between flies and human are attributed to the shared biochemistry behind protein-DNA contacts, then what drives the profound differences in TF binding stability between species? One possibility is the different developmental time points when fruit fly and mammalian TFs have been profiled. *Drosophila* TFs have almost always been examined at early developmental points; however, TFs active in mammalian embryonic stem cells show even greater divergence (Kunarso et al., 2010).

A stronger candidate would seem to be the different population genetics of flies and mammals, which have shaped dramatically different genome architectures along each lineage (González and Petrov, 2012; Lynch, 2007). *Drosophila* (with enormous breeding populations) have 15,000 genes covering 24 Mb of codons, located within a 120 Mb euchromatic genome, ∼80 Mb of which is under selective constraint (Halligan and Keightley, 2006; Stark et al., 2007; Keane et al., 2011). Mammals (with much smaller breeding populations) typically have 26,000 genes covering 45 Mb of codons, located within a 2,850 Mb euchromatic genome, 126 Mb of which is under selective constraint (Waterston et al., 2002; Lindblad-Toh et al., 2011; Ponting and Hardison, 2011). In other words, on average, every mammalian gene has about the same number of constrained noncoding regulatory bases as a *Drosophila* gene, but in mammals, they are spread across twenty times more euchromatic DNA that is not under obvious selective constraint.

Based on Lin and Riggs (1975), to compensate for dilution of functional, noncoding DNA, a corresponding increase in regulatory protein in the nucleus would be required in order to fully occupy functional TF binding sites, simultaneously resulting in many more nonfunctional sites. This increase in (nonfunctional) TF binding site numbers thus potentially explains the two major discrepancies between flies and mammals. First, because eukaryotic TF binding occurs over relatively narrow occupancy ranges (10–100-fold enrichments) (Biggin, 2011), the 20-fold increase in the number of potential TF binding sites per gene in mammals could be masking the simple intensity-function connection observed in *Drosophila* in part by complicating attempts to associate TFBRs with regulatory target genes. Second, the presence of 20-fold more potential TF binding locations could both facilitate migration of functionality between nearby sites as well as explain the rapid gain and loss of specific TF sites observed in closely related mammals.

In sum, our results confirm that the subtle quantitative differences in TF binding between species of mammals (like flies) are very likely the result of protein-DNA biophysics that has long been investigated. In contrast, the accumulation of qualitative gains and losses of TF binding between species (slower in flies and faster in mammals) appears to reflect the structure of their respective genomes, as determined by population genetics.

## Experimental Procedures

Experimental and computational procedures, including ChIP-seq, mouse genome sequencing, interspecies TF binding analysis, and knockout mouse functional analyses, were performed as detailed the Supplemental Information.

## Accession Numbers

All data have been deposited in ArrayExpress with accession numbers E-MTAB-1414 for mouse and E-MTAB-1415 for rat.

## Supplemental Information

Document S1. Extended Experimental Procedures

Document S2. Article plus Supplemental Information
