# Supplementary material for: Cooperativity and Rapid Evolution of Cobound Transcription Factors in Closely Related Mammals
Source: Cell. 2013 Aug 1;154(3):530–40. doi: 10.1016/j.cell.2013.07.007 (PMC3732390; doi:10.1016/j.cell.2013.07.007)
Supplement: Document S1. Extended Experimental Procedures [file mmc1.pdf]

Cooperativity and Rapid Evolution of Cobound Transcription Factors in Closely Related Mammals

Klara Stefflova, David Thybert, Michael D. Wilson, Ian Streeter, Jelena Aleksic, Panagiota Karagianni, Alvis Brazma, David J. Adams, Iannis Talianidis, John C. Marioni, Paul Flicek, and Duncan T. Odom

Extended Experimental Procedures

Supplemental Methods 1. Determination of TF-Bound Regions in Five Closely Related Mammals (p. 3)

SM1.1. Sample Preparation for ChIP-Seq (p. 3)
SM1.2. Mouse, Human, and Rat Genome Sequences Used (p. 3)
SM1.2.1. A/J, CAST/EiJ, and SPRET/EiJ SNV and Small Indel Calling (p. 4)
SM1.2.2. Caroli/EiJ Genome SNV and Small Indel Calling (p. 4)
SM1.2.3. Genome Alignment, the Reference Coordinates System, and Gene Annotation (p. 5)
SM1.3. TFBR Calling Pipeline Overview (p. 5)
SM1.4. TFBR False Discovery Estimation (p. 6)
SM1.5. De Novo Motif Discovery and Motif Search (p. 6)

Supplemental Methods 2. The Accumulation of Differences between TF Binding in Different Mouse Species Corresponds with Interspecies Evolutionary Distance (p. 7)

SM2.1. Qualitative Estimation of TF Binding Divergence across Species (p. 7)
SM2.2. Control of Threshold Effect on TF Binding Divergence Calculation (p. 7)
SM2.3. Estimation of TF Binding Divergence Rate across Evolutionary Time (p. 8)
SM2.4. Divergence Rate Calculation using only the Portion of the Mouse Genome Alignable to Rat (p. 8)
SM2.5. TF Binding Intensity Correlation among Individual Mice (p. 8)
SM2.6. TF Binding Intensity Correlation among Mouse Species (p. 9)
SM2.7. Assessing Genome Assembly Effects on TF Binding Intensity Correlation (p. 9)
SM2.8. Quantile Normalization of TFBR Intensities (p. 9)

Supplemental Methods 3. Differences in Bound Genetic Sequences Can Account for Only a Fraction of Differences in TF Binding in Closely Related Mammals (p. 10)

SM3.1. Definition of the Fraction of TFBRs with Sequence Variation in Bound Motifs among Species (p. 10)
SM3.2. Relationship between Binding Intensity Difference and SNV for Each Motif Position (p. 10)
SM3.3. Definition of the Fraction of TFBR with Sequence Variation among Species (p. 11)
SM3.4. TFBR Classification in Three Categories of TF Occupancy Conservation (p. 11)
SM3.5. SNV Density Distribution Analysis (p. 12)

SM3.6. GERP Score Distribution Analysis (p. 12)

Supplemental Methods 4. TF Binding in Combinatorial Clusters Evolves Coordinately (p. 13)

SM4.1. Identifying the Clusters of TF Combinatorial Binding (p. 13)
SM4.2. Coevolution of Cobound TFs (p. 13)

Supplemental Methods 5. TF Binding Intensities within Clusters Coevolve (p. 14)

SM5.1. Intensity Covariation of a Pair of Cobound TFs (p. 14)

Supplemental Methods 6. A Large Core Set of TF Binding Intensities Is Evolutionarily Stable across All Five Mouse Species (p. 14)

SM6.1. Relationship between TFBR Intensity and TFBR Conservation across Mouse Species (p. 14)
SM6.2. Correlation between the Conservation and Multiplicity of TF Binding in TFBRs (p. 15)
SM6.3. Frequency of SNV Occurrence in Motifs of Different TFBR Conservation Classes (p. 15)
SM6.4. Reconstruction of Ancestral TF Binding Profiles (p. 16)
SM6.5. TF Binding Intensity Class Definition (p. 16)
SM6.6. Classification of TFBRs by Evolutionary Behaviour (p. 17)
SM6.7. Estimation of Random Expectation (p. 18)
SM6.8. Intensity Assessment of TF Binding in Clusters (p. 18)
SM6.9. Identification of TFBRs Near Target Genes (p. 18)
SM6.10. Functional Enrichment of TFBRs (p. 19)
SM6.11. Genomic Distribution of TF Binding Conservation Categories (p. 19)
SM6.12. ChIP Intensity and Sequence Conservation Comparison of TF Binding Found near Target Genes (p. 20)

Supplemental Methods 7. The Genetic Deletion of a Single TF Has a Direct Effect on the Stability of the Remaining TFs within a Cobound Cluster (p. 20)

SM7.1. TFBR Identification from CEBPA and HNF4A KO Mouse ChIP-Seq Experiment (p. 20)
SM7.2. Testing the Cooperativity of TFs Bound within the Same Cluster Using HNF4A and CEBPA KO Mice (p. 21)
SM7.3. Analysis of Cluster Conservation in CEBPA and HNF4A KO Mice (p. 22)
SM7.4. SNV-Targeted Analysis of Module Instability (p. 22)

References (p. 23)

Supplemental Methods 1. Determination of TF-Bound Regions in Five Closely Related Mammals

SM1.1. Sample Preparation for ChIP-Seq

For the wild type mice, livers were obtained post-mortem from nutritionally unstressed males of 3-6 months of age (**Figure S1A**), maintained at the University of Cambridge, CRUK – Cambridge Institute under the auspices of a UK Home Office license. We performed chromatin immunoprecipitation experiments followed by high-throughput sequencing (ChIP-seq) on formaldehyde cross-linked livers from each of the five mouse species for at least two individuals (biological replicates), as previously described (Schmidt et al. 2009, see **Figure S1A**). The chromatin immunoprecipitation was performed using the following antibodies: CEBPA antibody sc-9314 (Santa Cruz), HNF4A antibody ARP31946 (Aviva Biosystems), and FOXA1 antibody ab5089 (Abcam). The immunoprecipitated material was end-repaired, A-tailed, ligated to single-end sequencing adapters, amplified by 18-cycles of PCR using Primer 1.1 and Primer 2.1, and size selected (200-300 bp), followed by single-end 36bp sequencing on an Illumina Genome Analyzer II, according to the manufacturer's instructions.

For the ChIP-seq in rat, we used the data under Array Express accession number E-MTAB-1415, and for corresponding human data, we used the HNF4A and CEBPA chip-seq data from (Schmidt et al. 2010), deposited under E-TABM-722.

Liver-specific CEBPA-null mice were generated by CRE-loxP-mediated excision of the entire coding region of the CEBPA gene by crossing CEBPA-floxed mice (Inoue et al., 2004) with mice carrying the albumin-*Cre* (AlbCre) transgene. Full inactivation occurred between postnatal days 25-30, and the experimental analyses reported here were performed between postnatal days 60-90.

Liver-specific HNF4A null mice were generated by inducible CRE-loxP-mediated excision of exons 4 and 5 of the *HNF4A* gene. To this end, *HNF4A*$^{lox/lox}$ (Hayhurst et al. 2001) mice were crossed with *TTR-Cre ind* (Tannour-Louet et al. 2002) mice. One-month-old *HNF4A*$^{lox/lox}$/ *TTRCre ind* mice were intraperitoneally injected with 2 mg/20 g/day of tamoxifen for 5 consecutive days. Analysis was performed at postnatal day 45. The same ChIP-seq protocol as described above was implemented for both CEBPA-null and HNF4A-null mouse livers.

SM1.2. Mouse, Human, and Rat Genome Sequences Used

Three genomes used in this study were previously published reference genomes – NCBI37 for the C57BL/6J mouse strain (Waterson et al. 2002), GRCh37 for the human reference genome and RGSC 3.4 for rat (Gibbs et al. 2004). Operational genome sequences were constructed for the four mouse species using single nucleotide variants (SNVs) and small indels called from whole genome sequencing (WGS) data mapped to the NCBI37 mouse reference genome (details below). The genome of the Caroli/EiJ mouse species was sequenced specifically for this study with 15-fold coverage of short-read sequences, while the sequence reads for A/J, CAST/EiJ and SPRET/EiJ were previously published (Keane et al 2011; ftp://ftp-mouse.sanger.ac.uk/current_bams).

SM1.2.1 - A/J, CAST/EiJ, and SPRET/EiJ SNV and Small Indel Calling

NGS reads from *A/J, CAST/EiJ and SPRET/EiJ* was mapped against the NCBI37 reference mouse genome with MAQ using default parameters. Then, SNVs and small indels were called using SAMtools version 0.1.7 with a consensus quality score above 30 for SNVs and 50 for small indels. We called the following number of SNVs and indels, respectively: 4,134,834 SNVs and 574,035 indels for A/J; 17,324,491 and 1,997,423 for CAST/EiJ and 33,970,867 and 3,599,092 for SPRET/EiJ.

SM1.2.2 - Caroli/EiJ Genome SNV and Small Indel Calling

For the *Mus caroli* (Caroli/EiJ), genomic DNA was sequenced with an effective coverage of 15x - 562,423,956 reads of 108 nt length, distributed in 10 libraries of short-range paired-end reads. Due to the higher evolutionary distance between the C57BL/6J and Caroli/EiJ mice, we used a different strategy to call SNVs and small indels than was used for A/J, Castaneus, or Spretus. Caroli SNVs and small indels were defined by a consensus of variant calling approaches based on a combination of WGS data mapping to a reference genome and WGS *de novo* genome assembly.

When Caroli/EiJ WGS data was mapped to the mouse reference genome, we used BWA with default parameters. In each resulting mapped library, we removed duplicate reads and then merged the 10 libraries into one unique BAM file. We then called three different datasets of SNVs using three different SNV callers: mpileup from SAMtools V0.1.18 (Li et al 2009), GATK v1.4 (McKenna et al. 2010) and freebayes v 0.8.4 (Garrison et al 2012). We called a fourth dataset of SNV based on the *de novo* assembly tool CORTEX v1.0.5 using the bubble calling approach (Iqbal et al. 2012).

The Caroli/EiJ SNV datasets contain 66,270,264 SNV from mpileup, 78,344,640 from GATK, 41,697,875 from freebays and 50,526,604 from the *de novo* assembly approach. We defined our final dataset of 67,363,757 SNVs as those called by at least two of the four approaches. The small indels were called from the WGS data mapped to the reference genome using *dindel* v1.01 (Albers et al 2011) with default parameters, leading to a dataset of 4,133,396 small indels.

SM1.2.3 - Genome Alignment, the Reference Coordinates System, and Gene Annotation

Based on indels called in the different mouse species, we constructed a mapping table representing a pairwise genome alignment between the different mouse genomes (i.e. A/J; CAST/EiJ; SPRET/EiJ; Caroli/EiJ) and the NCBI37 mouse reference. To compare mouse, human, and rat ChIP-seq datasets, we used the BLAST-Z alignment available in Ensembl v59. We used the NCBI37 mouse genome as a reference for comparing datasets from the different species. To annotate the different mouse genome sequences, we projected the coordinates of the v59 Ensembl gene annotation from the NCBI37 mouse reference genome to their corresponding operational genome sequence using the mapping tables.

SM1.3. TFBR Calling Pipeline Overview

In order to perform a high-accuracy comparison of TFBRs between closely-related mouse species, we developed an approach that identifies high confidence TFBRs reproducible between a pair of biological replicates of ChIP-seq data. We first mapped the ChIP-seq reads of each sample independently to their corresponding genome using BWA (Li et al. 2009) with default parameters. Then, for each factor and each species, we called TFBR with SWEMBL (Wilder et al. in preparation; http://www.ebi.ac.uk/~swilder/SWEMBL/) using input genomic DNA as to correct the effects of genome repeats and open chromatin on the experiment. The stringency level of SWEMBL (defined by the gradient function R in SWEMBL) was set both to maximize the number of TFBRs and to maximize the ability of the sequences obtained on input DNA to remove artefact regions called as peaks. The latter was done by analysing the fraction of Input peaks present in both Input and ChIP-seq samples: we defined as threshold the value of R parameter for which the rate of Input peaks shared with ChIP-seq data began to decrease dramatically. We obtained a typical R value of 0.005 for each replicate, and 0.004 for the dataset made by pooling two replicates.

For each factor in each species, we called three sets of TFBR: one from each replicate, and one from a pooled dataset of mapped reads from both replicates. The final set of TFBR selected was the intersection of these three datasets, in order to exclude regions not reproduced in both replicates. To do so, we selected the TF binding region from the pool dataset where homologous region in the two other replicates overlap with by at least one base pair. This approach accounts for both technical and biological variability.

SM1.4. TFBR False Discovery Estimation

To estimate the rate of false positive calls from our ChIP-seq data, we used 2 mock ChIP replicates (IgG; Protein G beads without a specific antibody incubated with cell lysates) and 6 input DNA replicates (whole cell extract). The input DNA controls for the effects of genome repeats and open chromatin on the experiment, while the mock replicates additionally account for the variability caused by nonspecific binding to the Protein G beads. We called peaks in all possible pairs of input samples corrected by other input samples and pairs of mock samples corrected by other input samples in order to estimate the average number of false positives TFBR peaks in each ChIP-seq sample.

For the peaks from the mock sample, we performed the same TFBR calling procedure described in section **SM1.3** –regions were called for the two individual replicates, as well as the pooled sample, and the intersection of all 3 sets of regions was taken as the final result. We also called peaks in pairs of input replicates with the same procedure described in **SM1.3**.

We identify an average of 118 peaks (with a minimum of 15 and maximum of 359) using the input DNA sample, and an average of 210 peaks (with a minimum of 137 and maximum of 369) in the mock sample. Given the high number of peaks called in the ChIP-seq replicates (more than 40,000 see **Figure S1C**), we estimate the number of falsely discovered TFBRs due to open chromatin or non-specific binding to the Protein G beads to be less than1% of the total peaks called in a standard ChIP experiment.

SM1.5. De Novo Motif Discovery and Motif Search

For motif discovery, we used MEME (Timothy, et al. 2006) with default settings. For each species, we performed motif discovery on both the top and bottom 2000 TFBRs ranked by intensity where intensity is defined as the number of reads mapped to a given TFBR. The bottom 2000 TFBRs were used to verify whether the same motifs found in the high intensity TFBRs

were also present in the low intensity ones, thus verifying the likelihood of the low intensity TFBRs being direct protein-DNA interactions. We selected the sequence of +/-25 base pairs centred on the identified summit of the peak as our input for motif discovery.

To identify the motif of each TRBR, the species-specific position weight matrices (SSPWM) from the 2000 top TFBRs dataset were scanned against the bound regions using the TFBS Perl modules (Lenhard et al. 2002) with a relative threshold of 0.8. When multiple words associated with an SSPWM were found in the binding region, we selected the word closest to the summit (**Figure S1D**).

Supplemental Methods 2. The Accumulation of Differences among TF Binding in Different Mouse Species Corresponds with Interspecies Evolutionary Distance

SM2. Qualitative Estimation of TF Binding Divergence across Species

To assess the similarity of TF binding profiles between different mice in terms of strict presence or absence of TF binding, we defined TFBRs (identified in SM1.3) as shared between two mouse species when the genomic location of ChIP-seq binding regions in two species overlaps by at least one base pair.

We defined as unshared a TFBR called in a species but which does not overlap with TFBRs called in the second species in pairwise comparisons.

Finally, we define as species-specific a TFBR present in one mouse species and not shared with *any* of the other four mouse species.

We calculated the fraction of shared TFBR between a pair of mouse species by calculating the mean of the fraction of TFBR in each species that are shared between them.

SM2.2 . Control of Threshold Effect on TF Binding Divergence Calculation

To assess if our thresholding of TF binding affected our calculated rate of binding divergence in a five-species TFBR comparison, we identified as ‘shadow TFBRs’ all loci bound by a TF in at least one mouse species, that were unshared with at least one of the other species with our standard peak calling parameters (**SM1.3**). For these shadow regions, we used

SWEMBL to call peaks that could be found with a more lenient threshold of peak calling. We then added to the initial dataset of TFBRs the new set of shadow TFBR and recalculated a new fraction of shared TFBRs between C57BL/6J and the other mouse species. This procedure was implemented with three different lenient thresholds. We used the values of R gradient function as follows: 0.004, 0.003, and 0.002. We found on average 20.04% of shadow regions containing TFBRs for CEBPA, 21.30% for HNF4A and 8.78% for FOXA1 with the most lenient condition (R=0.002). We found almost no change in the calculated rate of divergence using this approach (**Figure S1E**).

SM2.3. Estimation of TF Binding Divergence Rate across Evolutionary Time

In order to calculate the rate of TF binding difference accumulation across evolutionary time, we performed a linear regression of the data points of the fraction of shared TFBR vs. the evolutionary time on a log scale. We removed the A/J data point, since its evolutionary distance (0 MY) is not suitable for display in log-space. For CEBPA and HNF4A we used the mouse/human and mouse/dog fraction of shared TFBRs calculated in (Schmidt et al. 2010) for the 80MY evolutionary distance; for CEBPA only, we used the mouse/opossum fraction of shared TFBR for the 180MY evolutionary distance. We then calculated the parameters of the fitted function (**Figure S2A**).

SM2.4. Divergence Rate Calculation Using Only the Portion of the Mouse Genome Alignable to Rat

To control for the potential effect of *Mus* lineage-specific large indels on the decay rate, we calculated the rate of divergence between the five mice by considering only the TFBR included in the C57BL/6J genomic regions alignable with the rat genome. We identified these regions by using the BLAST-Z alignment between mouse and rat available in Ensembl v59. The fraction of TFBRs outside of the alignable regions was less than 10% in all cases (7.9-9.2% of the CEBPA TFBR; 8-8.6% for HNF4A and 7.6-8% for FOXA1).

SM2.5. TF Binding Intensity Correlation between Individual Mice

In order to establish a baseline for inter-strain and inter-species comparisons, we compared the correlation of TF binding intensity between multiple individuals of the same strain.

We performed ChIP-seq for two additional biological replicates for CEBPA in C57BL/6J (final total of four biological replicates), as well as two additional replicates for HNF4A and FOXA1 in SPRET/EiJ (four biological replicates total in the final analysis). We then called TFBR using the approach described in **SM1.3**. We plotted the normalized intensity (**SM2.8**) of the two sets of reproducible TFBRs from all four individuals (Figure S2F) and calculated the Pearson correlation both for TFBRs shared between individuals and for all TFBRs.

SM2.6. TF Binding Intensity Correlation between Mouse Species

To study TF binding intensity correlation during evolution, we calculated the Pearson correlation of the normalized intensity (**SM2.8**) of TFBRs shared between a pair of species (**Figure S2F**). The sets of TFBRs used for the comparison are the ones defined with our peak calling pipeline approach (defined in **SM1.3**). We also calculated the Pearson correlation of the set including all TFBRs (both the unshared and the shared TFBRs) between a pair of species. We define the intensity of the orthologous unbound region as 0.

The correlation value calculated with only the shared TFBR dataset is shown in **Figure S2F** in the top left side of each plot, and the correlation value calculated with all of the TFBR is shown in brackets in the same region of the plot.

SM2.7. Assessing Genome Assembly Effects on TF Binding Intensity Correlation

To estimate the maximum possible contribution of error in the operational genome sequences used for each mouse species, we used the C57BL/6J ChIP-seq reads and mapped them to the genomes of each of the four other mouse species. We then identified the TFBRs as described in **SM1.3**, and calculated the Pearson correlation of overlapping TF binding intensity between C57BL/6J and each of the artificial datasets as described in **SM2.6** (**Figure S2G**).

SM2.8. Quantile Normalization of TFBR Intensities

In order to have comparable TFBR intensities between species we normalized the intensity value using a quantile normalization approach. We created a matrix where each column represents a particular TF in a particular species and the rows are genomic locations where a TFBR was called in at least one species using the procedure described in **SM1.3**. For each locus where a particular TF was not bound in particular species, we set at 0 the value of the intensity

for the particular TF in that particular species. We then normalized the value in this matrix by using the R function normalize.quantiles.

Supplemental Methods 3. Differences in Bound Genetic Sequences Can Account for Only a Fraction of Differences in TF Binding in Closely Related Mammals

SM3.1. Definition of the Fraction of TFBRs with Sequence Variation in Bound Motifs between Species

To assess whether sequence variation of the bound motif (or nearest surrounding sequences) can explain most of the differences in TF binding between species, we performed pairwise comparisons between C57BL/6J and the other mice, rat and human. We selected the set of C57BL/6J TFBRs with at least one motif found using the TFBS Perl modules using the same conditions and SSPWM as defined in the section **SM1.5**. When multiple matches for the same motif were found in the TFBR, we selected the one closest to the summit to assess the sequence variation. 41,442 binding regions for CEBPA, 59,305 for HNF4A and 49,586 for FOXA1 were used. For each pairwise comparison, we classified TFBRs into two categories depending on whether they were shared or not between C57BL/6J and the other species (see **SM2.1**). We then counted in each category the fraction of TFBRs with a sequence variation in any base of the motif. To define the sequence variation in the motif, we used the SNV datasets described in section **SM1.2.1** and **SM1.2.2**. For the comparison of mouse/rat and mouse/human the orthologous sequences of C57BL/6J were found in human and rat genomes using the 35 way eutherian mammals EPO multiple alignments available from Ensembl v64. We controlled for the potential uncertainty in the multi-species alignment between mouse and rat or human by using the BLASTZ-NET pairwise alignment in the same version of Ensembl (data not shown).

We defined the high information content positions as the 50% of motif position having the most information content. This definition resulted in the analysis of positions 2,3,5,8,9 of the CEBPA motif; positions 2,5,6,8,9,10 for HNF4A and positions 5,6,8,9,11 for the FOXA1 motif. The PWM used to calculate the information content was defined using a *de novo* motif discovery in the 2,000 most intense TFBR as defined in **SM1.5** and **Figure S3C**.

SM3.2. Relationship between Binding Intensity Difference and SNV for Each Motif Position

We selected the set of TFBRs obtained by pairwise comparison between C57BL/6J and all other mice that have an SNV in the TF binding motif except *Mus caroli*, which was excluded from this section's analysis. This set of TFBRs includes both the species-specific and shared TFBRs between C57BL/6J and the other species. 17,538 binding regions were assessed for CEBPA, 22,690 for HNF4A and 18,410 for FOXA1.

For each TFBR we calculated the change in intensity between the orthologous TFBRs from the two species, defined as $\mathbf{\Delta}_{\text{intensity}}$ = Intensity $_{\text{C57BL/6J}}$ - Intensity $_{\text{other species}}$, where Intensity $_{\text{C57BL/6J}}$ is the intensity of the binding region in C57BL/6J mouse and Intensity $_{\text{other species}}$ is the intensity of the TFBR in the other species. We assigned a "0" intensity when a TFBR had no TF binding event in one of the two species.

For each position within the motif, we plotted the difference in intensity associated with a base change in that position (X→ T, X→A, X→G, X→C; where X can be any other base (**Figure S3D**). Finally, we calculated the Pearson correlation between information content and the mean of the absolute value of the delta intensity associated with an SNV in this position (**Figure S3E**).

SM3.3. Definition of the Fraction of TFBR with Sequence Variation between Species

To assess whether sequences outside the central TF binding motif under the ChIP signal had genetic variation that could help explain TF binding differences between species, we used a very similar approach to that used in **SM3.1**. We used the prior sets of TFBRs defined as shared or unshared between mouse species and counted in each category the fraction of TFBRs having at least one sequence variant in the region of +/-150 bp from each peak summit.

SM3.4. TFBR Classification in Three Categories of TF Occupancy Conservation

To link TF binding occupancy differences with SNVs in the TFBRs, we classified our TFBRs using pairwise comparison between C57BL/6J and the other mice into three categories: (i) <u>shared TFBR with conserved intensity</u>, (ii) <u>shared TFBR with altered intensity,</u> and (iii) <u>unshared TFBR</u>. We use the same definition of shared and unshared TFBR as previously defined in **SM2.1**.

To classify the peak intensity as either the same or different in another species, we assumed that peaks in biological replicates from the same strain that are in the same location as defined in **SM1.3** (i.e. replicated peaks) have the same true intensity. We then calculated an empirical distribution of the observed intensity differences for these replicated peaks (i.e. a distribution of the observed intensity differences arising from peaks with the same true intensity). From this distribution we empirically defined thresholds above which two intensities were different with an FDR of 0.05. We used the replicated data sets described in section **SM2.5** meaning that for the factor CEBPA, we used replicated peaks from C57BL/6J to define the empirical distribution and for HNF4A and FOXA1, we used replicated peaks from SPRET/EiJ. We found a delta intensity threshold of 212.42 for CEBPA, of 142.28 for HNF4A and 183.28 for FOXA1.

Depending on the pair of species, we used sets of 14,510 to 31,482 binding regions in the category “shared TFBR with conserved intensity”; 1,622 to 4,810 binding regions in the category “shared TFBR with altered intensity”, and 8,906 to 22,590 binding regions in the category “unshared TFBR” for CEBPA. For HNF4A the sets ranged from 13,359 to 37,286 binding regions in the category “shared TFBR with conserved intensity”, 3,331 to 8,499 binding regions category “shared TFBR with altered intensity”, and 25,778 to 31,519 in the category “unshared TFBR”. Finally for FOXA1, there were 12,978 to 35,989 binding regions in the category “shared TFBR with conserved intensity”, 6,405 to 12,978 binding regions in the category “shared TFBR” with altered intensity and 10,254 to 29,385 in the category “unshared TFBR”.

SM3.5. SNV Density Distribution Analysis

For each category of TF occupancy conservation defined above (**SM3.4**), we used the dataset of SNVs defined in **SM1.2.1** and **SM1.2.2** to look at the density of SNVs in a region of +/- 1000 bp around the TFBR summit. We then calculated the Z-score of the SNV density, based on the mean and the standard deviation of the SNV density of the whole region, and plotted the smoothed SNV density using a sliding window approach (+/-7 bp) (**Figure S3G**).

SM3.6. GERP Score Distribution Analysis

We looked at the conservation of sequence in relation to the TF binding intensity. We used the same classes of TFBRs as described above (**SM3.4**), and we calculated and plotted the

mean of the Genomic Evolutionary Rate Profiling (GERP) score (Cooper et al 2005) for each position +/- 1000 bp around the summit. The p-values for the differences between the three TFBR classes were obtained by comparing the GERP score distributions from the regions +/- 25 bp around the summit using a t-test (**Figure S3G**).

Supplemental Methods 4. TF binding in Combinatorial Clusters Evolves Coordinately

SM4.1. Identifying the Clusters of TF Combinatorial Binding

We identified clusters of TF binding by selecting all groups of TFBRs with a maximum distance of 300 bp between two consecutive summits. We categorised these clusters into different groups: The 1TF clusters (singletons) contain only one TF binding event, the 2TF clusters have binding events with two different TFs, and the 3TF clusters have binding events with all three TFs. In order to avoid ambiguity during combinatorial binding analysis we ignored the clusters with more than one binding event of the same TF. The clusters with more than one binding event of the same TF represent between 7.15% and 13.45% of the regions called depending on the species considered.

SM4.2. Coevolution of Cobound TFs

We analyzed whether combinatorial TF binding evolves co-ordinately by anchoring our analysis on a single TF within the context of a combinatorially bound cluster. Here, we will describe how we analyzed the pattern of conservation and divergence of clusters in C57BL/6 which contain FOXA1 when interrogated in Mus castaneus; identical analyses were performed for each TF and species-pair in turn.

We began by subdividing the 1TF, 2TF, and 3TF clusters containing FOXA1 by whether FOXA1 binding is shared or unshared in Mus castaneus (**Figure 3**, green and grey shading, respectively). Note that we did not consider the cases where there were gains of TF binding in Mus castaneus.

For each combinatorial class (e.g. 2TF and 3TF) of FOXA1 binding event with shared FOXA1 binding, we then asked whether co-bound CEBPA and/or HNF4A were shared or unshared in Mus Castaneus. If all were shared, then these 2TF/3TF clusters were labelled totally

shared. If one or more were absent, then these 2TF and 3TF clusters were labelled partially shared.

For each combinatorial class (e.g. 2TF and 3TF) of FOXA1 binding event with unshared FOXA1 binding, we then asked whether co-bound CEBPA and/or HNF4A were shared or unshared in Mus Castaneus. If one or more of the co-bound TFs were shared, then these 2TF/3TF clusters were labelled partially unshared. If all co-bound TFs were absent, then these 2TF and 3TF clusters were labelled totally unshared.

Note that for the 1TF category we can only observe total unshared events since no other factor is associated. We performed this analysis for all three TFs and all four possible pairwise comparisons among C57BL/6J and A/J; CAST/EiJ; SPRET/EiJ; Caroli/EiJ (**Figure 3** and **Figure S4C**).

Supplemental Methods 5. TF Binding Intensities within Clusters Coevolve

SM5.1. Intensity Covariation of a Pair of Cobound TFs

In order to see whether TFs binding in the same region show a co-variation of binding intensity, we looked at the variation of intensity in pairs of co-bound TFs using a pairwise comparison between C57BL/6J and each of the other mouse species. We used the normalized number of reads as the measure of intensity. We considered within the 2TF and 3TF clusters all pairs of TFs that are shared between two species. For example, if a 3TF module in C57BL/6J is shared with a 2TF module in SPRET/EiJ consisting of FOXA1 and HNF4A, we considered only the intensity variation of the two TF binding events occurring in both species. We quantile normalized the intensities of all pairs of TFs, and then calculated the intensity differences for each of the TFs and plotted it, adding a Pearson correlation (**Figure 4** and **Figure S4D**).

Supplemental Methods 6. A Large Core Set of TF Binding Intensities Is Evolutionarily Stable across All Five Mouse Species

SM6.1. Relationship between TFBR Intensity and TFBR Conservation across Mouse Species

To look at the relationship between TF binding intensity and the conservation of TF binding across the mouse species, we classified the TFBRs by the number of species in which

they were shared (**Figure S5B**). We compared the set of C57BL/6J TFBRs (called with the peak-calling pipeline defined in **SM1.3**) to the other four mouse species. When a TFBR was present only in C57BL/6J we classified it in the category "1 species"; when a C57BL/6J TFBR was shared with one other species, we classified in the category "2 species" and so forth. For each category, we plotted the number of reads normalized by quantile normalization (see **SM2.8**).

SM6.2. Correlation between the Conservation and Multiplicity of TF Binding in TFBRs

In order to assess if the more conserved TFBRs are more likely to be part of a cluster of TF binding, we used all C57BL/6J TFBRs categorized into the 5 conservation categories (**SM6.1**) and plotted the fraction of TFBRs belonging to a cluster (defined as 2TF or 3TF in **SM4.1**) (see **Figure S5C**).

SM6.3. Frequency of SNV Occurrence in Motifs of Different TFBR Conservation Classes

In order to determine if TF binding sites more comprehensively across mice were more robust to sequence changes, we calculated the fraction of TFBR with an SNV in the motif and normalised this by the total number of shared binding sites for each conservation class (i.e. shared between two species, shared between three species, etc). This calculation was done for all pairwise comparisons of species and for all TFs with similar results.

As a specific example of the analysis, the results for the C57BL/6J SPRET/EiJ comparison are plotted in **Figure S5D** and were calculated by selecting the set of C57BL/6J TFBRs that are both shared with SPRET/EiJ and have an SNV in the motif. We then classified this set of TFBRs by the number of species in which they are shared. We calculated the enrichment E_i for each conservation class i by counting the fraction C_i of shared TFBRs with an SNV in the motif in the class (if i=2 it means that the TFBR is shared between C57BL/6J and one other species; if i=3 it means that the TFBR is shared in C57BL/6J and two other species, and so on) and dividing the C_i by the fraction F_i of the C57BL/6J TFBR belonging to the conservation class i , regardless of whether the TFBR contained an SNV. Finally, for each class we plotted the value $E_i = C_i/F_i$. The same analysis for followed for all pairwise comparisons..

SM6.4. Reconstruction of Ancestral TF Binding Profiles

We used the general parsimony approach (see (Swofford et al 2001) and (Berlocher 1997)) to reconstruct the ancestral TF binding profile of each internal ancestor as well as the last common ancestor of all study species. For a maximum parsimony analysis, we considered the intensity of TF binding as a character state. This approach tries to minimize within the evolutionary tree the cost of the transformation of character state to another between each internal ancestor and the observed data. A character is an attribute along which taxa are observed to vary and, in this case, we used the TF binding intensity class defined below (**SM6.5**) as a character. Therefore, the algorithm inferred the ancestral binding profile requiring the least cost change in intensity class to obtain the descendant genome binding profiles.

The model of transition cost of one intensity class to another is $C_{ij}= |j-i|$; where i denotes the i-th intensity class, and j the j-th intensity class. For example, a transition of a common ancestor with intensity class 3 to its direct descendent with intensity class 4 is $C_{34}=|3-4|=1$. When multiple most parsimonious evolutionary scenarios were defined for one locus, all of them were kept and evaluated as part of the downstream analysis (see **SM6.6** below).

SM6.5. TF Binding Intensity Class Definition

We classified the TFBRs in each species into 11 classes, based on quantiles of intensity. Class 0 refers to a region with no TFBRs called, while classes 1 to 10 are associated with a gradual increase in intensity. The distribution of peak intensity in the datasets follows an inverse factorial-like distribution, and so had to be normalized in order to make the intensity variation comparable between the different classes. This normalization was performed as follows: within each species, the TFBRs were first ordered by intensity, and then divided into 120 quantiles (corresponding to the number 5!). These subgroups were then organised into 5 classes using a factorial framework. Class 5 is the highest intensity class, and contained a total of 1! of the 120 quantiles (i.e. just the 1 top quantile). Class 4 then contained 2! of the next quantiles (the next 2 highest intensity quantiles), class 3 contained 3! (the next 6 intensity quantiles) and so on, with class 1 containing all TFBRs that were not grouped into the first four classes. This was necessary since the high intensity TFBRs show much more variation than the low intensity ones, and classifying them in this way normalized for this variation.

In order to give greater resolution, the original 5 classes were further subdivided such that there were 10 total classes. This was again done using a factorial framework, but in order to

preserve the structure of the original 5 classes, rather than dividing the original data into 10 factorial classes, instead each of the existing 5 classes was split into 2, but using the factorial framework described above. For instance, class 5 became classes 9 and 10, where class 5 was first divided into 3 subgroups (that is, 2! subgroups); where the new class 10 contained the first subgroup with the most intense TFBR, whereas the new class 9 contained the remaining two subgroups with less intense TFBR. Each class has on average an intensity range (in normalized read counts) of 155.75 for CEBPA, 170.65 for HNF4A and 174.87 for FOXA1.

SM6.6. Classification of TFBRs by Evolutionary Behaviour

In order to study the trends of binding intensity change over evolutionary time, we used the common ancestor binding profiles reconstructed from the mouse species studied, using the parsimony approach described above. Only the TFBRs shared by all species were used for this analysis. The shared TFBRs were then classified into 3 classes – conserved, progressive and random. To do this, we used the 11 intensity classes described in **SM6.5**. The conserved TFBRs were defined as TFBRs changed by no more than one intensity class between the species in the evolutionary path from the last common ancestral TF binding locus to C57BL/6J. In other words, for an ancestor k with intensity I_k and its direct descendant $k+1$ with intensity I_{k+1} we considered their intensity as different if $| I_k - I_{k+1} | > 1$. The progressive class was defined as a TFBR that differed in direction consistently (either increasing or decreasing) by at least 2 classes, and that differed at least twice between the four ancestral states. The random class is analogous to the null hypothesis of binding that is not under progressive or stabilising constraint, and included all TFBRs with intensity that changed, but not progressively.

For the loci with more than one most parsimonious evolutionary scenario, we assign a class if more than 70% of the scenarios agreed on the same class of TF binding intensity evolution. Otherwise, the locus was called as undetermined.

On average the range of one intensity class defined in **SM6.5** is slightly below the threshold of intensity differences used to determine whether two TFBRs have different intensities, based on an FDR of 5% (see **SM3.4**). However, we required an intensity difference of two intensity classes to state that binding sites do not have conserved intensity. Thus, we increased the confidence that two TFBR truly have different intensities at the expense of missing some true sites with small differences. In order to control for effects of the discretisation of the continuous

intensity values into classes, we repeated the analysis using the original 5 classes, rather than 10 – doing so did not affect the results of the analysis. We also repeated the analysis using a definition of conserved TF binding intensity that required the TFBRs to not change intensity class ancestors (rather than allowing for a change to the nearest class) and this also did not change the outcome of the analysis when using either 10 or 5 intensity classes.

SM6.7. Estimation of Random Expectation

We calculated an estimate of the expected distribution of conserved, progressive and randomly evolving classes if the TF binding intensity value was randomly organized between species. The random expectation calculation for TFBRs classification was done using a permutation-based approach. For each species, the TFBRs intensities were randomly shuffled in order to break the orthology relationship between the TFBRs in each species. The ancestral TFBR profiles inferred from the new simulated datasets were then reclassified as described above. The same procedure was repeated a million times, and the median numbers of TF binding class were then taken as the random expectation. We tested the significance of the difference between observed and expected proportions of the three classes by calculating an empirical p-value from the simulations.

SM6.8. Intensity Assessment of TF Binding in Clusters

To look at the relationship between TF binding intensity and the number of co-bound partners in a cluster, we used the cluster definitions from **SM4.1**. For each factor, we classified the TFBR by whether it was included in a 1TF, 2TF or 3TF cluster, and then the distribution of the normalized intensity of each class of TFBR was plotted (**Figure S5A**).

SM6.9. Identification of TFBRs near Target Genes

In order to identify TFBRs that are more likely to be functional, we analysed a set of C57BL/6J TFBRs in the vicinity of known target genes. In the first step, we selected a gold standard set of 155 CEBPA target genes resulting from gene expression analysis of a CEBPA KO mouse, as defined in (Schmidt et al. 2010), and another gold standard set of 240 HNF4A target genes obtained from both knockout mouse and human data and defined in (Boj et al. 2009).

These genes are generally directly dependent on the presence of the TF for proper expression, and often have TF binding nearby.

To define the set of TFBRs near target genes, we selected all C57BL/6J TFBRs that fall between 10kb upstream of the transcription start site of a target gene and 10kb downstream of the end of a target gene. Using this definition, we identified a total of 630 CEBPA and 1,774 HNF4A TFBRs near target genes.

As a control category, we used the set of CEBPA TFBR and HNF4A TFBR that fall within the same windows near *all* protein coding genes based on the set of all 22,719 mouse protein-coding genes defined in Ensembl v64. The control category contains 30,474 CEBPA TFBR and 52,575 HNF4A TFBRs.

SM6.10. Functional Enrichment of TFBRs

We assessed the functional enrichment of genes near two different types of TFBRs (**Figure S5F-I**).

In the first analysis, the goal was to look at the relationship between TF occupancy conservation and functional categories of the regulated genes. For each factor, we used three sets of genes. The first set was defined by the TFBR near target genes (**SM6.9**), the second set was defined by the set of C57BL/6J species-specific TFBR (see definition in **SM2.1**), the last set of TFBRs was defined as the C57BL/6J TFBRs shared by all five mouse species that also have conserved intensity (see definition in **SM3.4**). We analyzed the three sets of TFBR with the web tool GREAT (McLean et al. 2010) using the default parameters and using as a background the whole set of C57BL/6J TFBRs.

In the second analysis, we classified the C57BL/6J TFBRs in five intensity categories by merging the five intensity classes originally defined in **SM6.5**. We then used the web tool GREAT to identify the functional enrichments of our five categories of TFBRs with default parameters and using as background the whole set of C57BL/6J TFBRs.

SM6.11. Genomic Distribution of TF Binding Conservation Categories

The goal of this analysis was to look at the genomic distribution of different conservation categories of TFBRs. The first category includes the whole set of C57BL/6J TFBRs described in **SM1.3**. The second category includes the union of the sets of TFBR called in all five mice with

our peak-calling pipeline described in **SM1.3**. The third category includes the C57BL/6J TFBRs that are present in all five species. Finally, the fourth category includes C57BL/6J TFBRs that are present in all five species and that have a conserved intensity (as defined in **SM3.1**). For each of these categories we represented in **Figure S5E** the number of TFBRs that are falling inside: (i) an exon and (ii) an intron of protein gene, (iii) a non coding RNA, (iv) an intragenic region, and finally (v) a promoter defined as the region starting from 10 kb upstream a transcription start site (TSS) of a gene and ending at the TSS. Genomic annotations were obtained from ENSEMBL version 59. The assessment of the significance of the genomic location distribution difference between these categories has been done with a Fisher exact test by comparing the distribution of the category of all C57BL/6J TFBRs with the other categories.

SM6.12. ChIP Intensity and Sequence Conservation Comparison of TF Binding Found near Target Genes

In order to see the relationship between functional TFBRs and intensity, we used for HNF4A and CEBPA three sets of C57BL/6J TFBRs, and compared their intensity distributions. The first set of TFBRs was the complete set of C57BL/6J TFBRs, as called with the peak-calling pipeline defined in **SM1.3**. The second set was the set of TFBRs near protein coding genes (e.g. within any protein coding gene body or within 10 KB of the 5' TSS or 3' UTR of any gene), and the third set was defined as the TFBRs near target genes (per **SM6.9)**. To assess the significance of the differences among these three categories, we performed a paired t - test. We also looked at the sequence conservation for these three categories of TFBR. For each category, we plotted the GERP score of the region of +/-150bp around the peak summit, and assessed the significance of the difference of these distributions by a t - test.

Supplemental Methods 7. The Genetic Deletion of a Single TF Has a Direct Effect on the Stability of the Remaining TFs within a Cobound Cluster

SM7.1.TFBR Identification from CEBPA and HNF4A KO Mouse ChIP-Seq Experiment

We identified TFBRs in liver tissues from CEBPA and HNF4A KO mice. The read mapping step was the same as described in **SM1.3** and we called peaks with SWEMBL with the R gradient function of 0.005 and using genomic DNA as an input.

SM7.2. Testing the Cooperativity of TFs Bound within the Same Cluster Using HNF4A and CEBPA KO Mice

To test the hypothesis that cooperativity contributes to the coevolution of combinatorial binding, we performed ChIP-seq experiments on mice genetically engineered to lack CEBPA and HNF4A genes. We looked at whether genetically removing one of the TFs within a cluster of TFs affected the binding of the two other TFs within that cluster (HNF4A and FOXA1 for the CEBPA knock out mice, and FOXA1 and CEBPA for the HNF4A KO mice).

To do this, we selected all the C57BL/6J 2TF and 3TF co-binding clusters in the wild type mouse (defined in **SM4.1**). Again, we did not consider in this analysis the regions where the same factor was present more than once in the same cluster in C57BL/6J as well as the regions where clusters with multiple TFBRs from the same factor were present in an orthologous region in at least one other mouse species. We then compared the overlap of a single replicate of HNF□□ and FOXA1 ChIP-seq TFBRs from the liver of a CEBPA knockout mouse, and a single replicate of FOXA1 and CEBPA ChIP-seq TFBRs from the liver of a HNF4A knockout mouse with combinatorial TF binding regions described above. The 2TF clusters that contained only HNF4A and FOXA1 were used as an internal control, representing the HNF4A and FOXA1 binding not affected by the CEBPA KO. This gave us the baseline of how much of the non-overlapping binding was due to inherent variability, and provided robustness to quality differences between samples. Similarly, the 2TF clusters containing only FOXA1 and CEBPA were used as an internal control for the HNF4A KO.

Additionally, we used the overlap of CTCF binding regions between the C57BL/6J (WT, wild type) mouse and the two KO mice as an external control of technical variability, since CTCF is rarely associated with the clusters of TF binding. For the CTCF from wild type C57BL/6J liver, we used the published data from (Schmidt et al 2010) with accession number ERR022291 and ERR022305. We called the TFBRs with SWEMBL R 0.005 using one replicate for each factor from the CEBPA and HNF4A KO mice.

In order to assess the statistical significance of the perturbation of the KO on TF binding, we did a Fisher exact test by comparing the overlap of TFBRs from KO mice with the different clusters of TF binding that may be affected by the KO and compared those to the control modules (**Figure S6.1**).

SM7.3. Analysis of Cluster Conservation in CEBPA and HNF4A KO Mice

In order to see how the conservation of clusters affects the robustness toward genetic perturbation, we selected all the C57BL/6J 2TF and 3TF co-binding clusters. We filtered out the regions where the same factor was present at least two times in the same cluster in C57BL/6J. We also removed the regions were these redundant clusters were present in an orthologous region in at least one other mouse species. We divided each type of cluster into different categories based on the level of conservation with the other four mouse species. The 'conserved' group had complete conservation between all five species, in the 'partial' group the combinatorial binding cluster was conserved, but some individual TF binding within it was lost, in the 'loss' group, the whole cluster was lost in at least one species, and in the 'unique' group, the cluster is only present in C57BL/6J. We then compared the overlap of HNF□□ and FOXA1 ChIP-seq TFBRs from the liver of a CEBPA KO mouse with these regions.

SM7.4 . SNV-Targeted Analysis of Module Instability

We tested whether genetic knockout of a TF is more likely to affect multi-TF clusters where mutations in the motif of the genetically deleted TF appear to be the only SNV that could account for the concurrent loss of *all* TF binding within the module in any other mouse species. We selected all 3TF regions present in C57BL/6J, and filtered out all clusters having more than one binding event of the same TF. We then performed a pairwise comparison of these regions with the other mouse species, and for each TF, we selected regions of complete cluster loss where an SNV appears in the motif for the factor we knocked out, but not in the motif of the two other factors.

For example, in the case of the HNF4A binding between C57BL/6J and CAST/EiJ, we selected (i) all 3TF clusters present on C57BL/6J and absent in CAST/EiJ that (ii) have a SNV in the HNF4A motif but no SNV in the CEBPA and FOXA1 motifs. Due to the SNV in the HNF4A motif, the loss of the entire cluster in CAST/EiJ is most likely attributed to the loss of HNF4A binding event. As a control, we also selected for each factor all 3TF regions having a SNV in the bound motif (and not in the motif of the two neighbours) but having lost only the factor with the SNV and not the neighbours. From this, we finally filtered out all the regions having at least two

binding regions of the same factor in CAST/EiJ mouse. We then calculated the fraction of these regions that overlap with the TF binding identified from the HNF4A KO mice.

Similar analysis was done for the CEBPA TF binding data and CEBPA KO mouse.

REFERENCES

Albers, C.A., Lunter, G., MacArthur, D.G., McVean, G., Ouwehand, W.H., and Durbin, R. (2011). Dindel: accurate indel calls from short-read data. Genome Res *21*, 961-973.

Bailey, T.L., N. Williams, C. Misleh, W. W. Li, "MEME: discovering and analyzing DNA and protein sequence motifs" *Nucleic Acids Research*, Vol. 34, pp. W369-W373, 2006.

Berlocher, S.H., and Swofford, D.L. (1997). Searching for phylogenetic trees under the frequency parsimony criterion: an approximation using generalized parsimony. Syst Biol *46*, 211-215.

Boj, S.F., Servitja, J.M., Martin, D., Rios, M., Talianidis, I., Guigo, R., and Ferrer, J. (2009). Functional targets of the monogenic diabetes transcription factors HNF-1alpha and HNF-4alpha are highly conserved between mice and humans. Diabetes *58*, 1245-1253.

Cooper, G.M., Stone, E.A., Asimenos, G., Green, E.D., Batzoglou, S., and Sidow, A. (2005). Distribution and intensity of constraint in mammalian genomic sequence. Genome Res *15*, 901-913.

Garrison et al., (2012) ARXIV pre-release at http://arxiv.org/abs/1207.3907

Gibbs, R.A., Weinstock, G.M., Metzker, M.L., Muzny, D.M., Sodergren, E.J., Scherer, S., Scott, G., Steffen, D., Worley, K.C., Burch, P.E*., et al.* (2004). Genome sequence of the Brown Norway rat yields insights into mammalian evolution. Nature *428*, 493-521.

Hayhurst, G.P., Lee, Y.H., Lambert, G., Ward, J.M., and Gonzalez, F.J. (2001). Hepatocyte nuclear factor 4alpha (nuclear receptor 2A1) is essential for maintenance of hepatic gene expression and lipid homeostasis. Mol Cell Biol *21*, 1393-1403.

Iqbal, Z., Turner, I., and McVean, G. (2012). High-throughput microbial population genomics using the Cortex variation assembler. Bioinformatics.

Kasowski, M., Grubert, F., Heffelfinger, C., Hariharan, M., Asabere, A., Waszak, S.M., Habegger, L., Rozowsky, J., Shi, M., Urban, A.E*., et al.* (2010). Variation in transcription factor binding among humans. Science *328*, 232-235.

Keane, T.M., Goodstadt, L., Danecek, P., White, M.A., Wong, K., Yalcin, B., Heger, A., Agam, A., Slater, G., Goodson, M*., et al.* (2011). Mouse genomic variation and its effect on phenotypes and gene regulation. Nature *477*, 289-294.

Lenhard, B., and Wasserman, W.W. (2002). TFBS: Computational framework for transcription factor binding site analysis. Bioinformatics *18*, 1135-1136.

Li, H., and Durbin, R. (2009). Fast and accurate short read alignment with Burrows-Wheeler transform. Bioinformatics 25, 1754-1760.

McKenna, A., Hanna, M., Banks, E., Sivachenko, A., Cibulskis, K., Kernytsky, A., Garimella, K., Altshuler, D., Gabriel, S., Daly, M., et al. (2010). The Genome Analysis Toolkit: a MapReduce framework for analyzing next-generation DNA sequencing data. Genome Res 20, 1297-1303.

McLean, C.Y., Bristor, D., Hiller, M., Clarke, S.L., Schaar, B.T., Lowe, C.B., Wenger, A.M., and Bejerano, G. (2010). GREAT improves functional interpretation of cis-regulatory

regions. Nat Biotechnol *28*, 495-501.
Reddy, T.E., Gertz, J., Pauli, F., Kucera, K.S., Varley, K.E., Newberry, K.M., Marinov, G.K., Mortazavi, A., Williams, B.A., Song, L*., et al.* (2012). Effects of sequence variation on differential allelic transcription factor occupancy and gene expression. Genome Res *22*, 860-869.
Schmidt, D., Schwalie, P.C., Wilson, M.D., Ballester, B., Goncalves, A., Kutter, C., Brown, G.D., Marshall, A., Flicek, P., and Odom, D.T. (2012). Waves of retrotransposon expansion remodel genome organization and CTCF binding in multiple mammalian lineages. Cell *148*, 335-348.
Schmidt, D., Wilson, M.D., Ballester, B., Schwalie, P.C., Brown, G.D., Marshall, A., Kutter, C., Watt, S., Martinez-Jimenez, C.P., Mackay, S*., et al.* (2010). Five-vertebrate ChIP-seq reveals the evolutionary dynamics of transcription factor binding. Science *328*, 1036-1040.
Schmidt, D., Wilson, M.D., Spyrou, C., Brown, G.D., Hadfield, J., and Odom, D.T. (2009). ChIP-seq: using high-throughput sequencing to discover protein-DNA interactions. Methods *48*, 240-248.
Swofford, D.L., Waddell, P.J., Huelsenbeck, J.P., Foster, P.G., Lewis, P.O., and Rogers, J.S. (2001). Bias in phylogenetic estimation and its relevance to the choice between parsimony and likelihood methods. Syst Biol *50*, 525-539.
Tannour-Louet, M., Porteau, A., Vaulont, S., Vasseur-Cognet, M. 2002. A tamoxifen-inducible chimeric Cre recombinase specifically effective in the fetal and adult mouse liver. *Hepatology* **35:** 1072-81.
Waterston, R.H., Lindblad-Toh, K., Birney, E., Rogers, J., Abril, J.F., Agarwal, P., Agarwala, R., Ainscough, R., Alexandersson, M., An, P*., et al.* (2002). Initial sequencing and comparative analysis of the mouse genome. Nature *420*, 520-562.
